# The endangered Florida pondweed (*Potamogeton floridanus*) is a hybrid: Why we need to understand biodiversity thoroughly

**DOI:** 10.1371/journal.pone.0195241

**Published:** 2018-04-02

**Authors:** Zdeněk Kaplan, Judith Fehrer, Veronika Bambasová, C. Barre Hellquist

**Affiliations:** 1 The Czech Academy of Sciences, Institute of Botany, Průhonice, Czech Republic; 2 Department of Botany, Faculty of Science, Charles University, Prague, Czech Republic; 3 Department of Biology, Massachusetts College of Liberal Arts, North Adams, Massachusetts, United States of America; Technical University in Zvolen, SLOVAKIA

## Abstract

Thorough understanding of biodiversity is a fundamental prerequisite for biological research. A lack of taxonomic knowledge and species misidentifications are particularly critical for conservation. Here we present an example of *Potamogeton floridanus*, the Florida Pondweed, an endangered taxon endemic to a small area in the Florida panhandle, whose taxonomic status remained controversial for more than a century, and all previous attempts to elucidate its identity have failed. We applied molecular approaches to tackle the origin of the mysterious taxon and supplemented them with morphological and anatomical investigations of both historical herbarium collections and plants recently collected in the type area for a comprehensive taxonomic reassessment. Sequencing of two nuclear ribosomal markers and one chloroplast non-coding spacer resulted in the surprising discovery that *P*. *floridanus* is a hybrid of *P*. *pulcher* and *P*. *oakesianus*, with the former being the maternal parent. The hybrid colony is currently geographically isolated from the distribution range of *P*. *oakesianus*. We show that previous molecular analyses have failed to reveal its hybrid identity due to inadequate nuclear DNA sequence editing. This is an example how the uncritical use of automized sequence reads can hamper molecular species identifications and also affect phylogenetic tree construction and interpretation. This unique hybrid taxon, *P*. *×floridanus*, adds another case study to the debate on hybrid protection; consequences for its conservation are discussed.

## Introduction

Endemic species, being confined to a small geographic area and usually relatively poor in individuals, can easily become endangered or extinct. Their populations are often rapidly declining or are on the verge of vanishing. These species are classified as ‘endangered’ and are justifiably in a priority focus of conservation biologists. They are often studied from various ecological or genetic aspects such as effective population size (e.g. [1,2]) and inbreeding depression (e.g. [3,4]), or to provide effective conservation and management measures (e.g. [5–7]). However, the need of a deep insight into their evolutionary origin and taxonomic position is sometimes underestimated, although exact assessment of all aspects of each species is necessary for use by conservationists in prioritizing their work.

The cosmopolitan genus *Potamogeton* (Potamogetonaceae) is the most species-rich genus of aquatic plants [8]. It includes about 72 species and at least 99 hybrids ([9], and Kaplan & Fehrer, unpublished data), with the centre of diversity in temperate regions of the Northern Hemisphere. High diversity [8], reduced morphology [10–12], extensive phenotypic plasticity [13], partitioning of genetic variation between rather than within populations [10, 14], polyploidy [15] and occurrence of numerous hybrids [8–9, 16] are the main sources of evolutionary and systematic complexity of this genus.

The general picture of species diversity in *Potamogeton* is relatively well known [8], and modern taxonomic revisions are available for several large areas, including Europe [17], Siberia [18], China [19], Malesia [20], Australia [21], North America [22] and the Neotropics [23]. On the other hand, there are many local forms that are poorly known and whose exact taxonomic identity is unclear (e.g. [15, 20, 24–31]). Whether these plants represent recently evolved taxa geographically confined to small areas, old species surviving in refugia and close to extinction, aberrant forms of widespread species, or interspecific hybrids cannot be easily addressed and requires further detailed investigation in each particular case.

Molecular methods can be used as an effective tool to reveal species relationships, to refine species delimitation in morphologically poorly differentiated complexes and to reveal hybrids masked under the phenotypic variation of pure species. Several molecular phylogenies focused on *Potamogeton* species assemblages of particular areas are available, e.g., for Japan [32], North America [33–34] or China [35–37]. The geographically broadest and most species-rich phylogeny to date is included in Kaplan et al. [15]. Ito et al. [38] have used a compilation of available sequence data to study some unclear South American samples. Recent studies on *Potamogeton* show that even in relatively well explored areas such as Europe or North America, the origin of the observed variation is poorly understood, and a great deal of the diversity is neglected and remains undetected [11–12, 39–40]. Molecular analyses have recently contributed to elucidate the systematic position of taxonomically uncertain local forms [15, 41], the discovery or exact identification of several entirely new hybrid combinations [11, 40, 42–46] and even confirmed the existence of a triple hybrid in *Potamogeton* [47].

*Potamogeton floridanus* Small is a unique taxon confined to a small area in the Florida Panhandle. Although it was first recorded by botanists already in 1886 (collected by A. H. Curtiss and recognized as “a peculiar form of *P*. *natans*” by Morong [48]) and validly described more than a century ago [49], it has always been considered a mysterious plant. Graebner [50] considered it to be doubtful in his monograph. Taylor [51] commented *P*. *floridanus* as “apparently an immature form” of *P*. *natans*. Bennett [52] suggested that it might be identical with *P*. *tepperi* A. Benn., a species described from Australia [53] and later recorded also from Asia [54–56]. Lacking any herbarium material for inspection, Hagström only listed *P*. *floridanus* in an index of *Potamogeton* names in his otherwise precise monograph [57]. The most detailed analysis of *P*. *floridanus* was given by Ogden [58], who described stem anatomy of the type collection and provided several hypotheses on its identity. He suggested that it might be a hybrid or a pronounced ecological form of *P*. *oakesianus* or *P*. *natans*. He mentioned various hybrid combinations with *P*. *pulcher*, *P*. *oakesianus* and *P*. *natans* as possible parents. However, he finally rejected involvement of these species for phytogeographical reasons and concluded that “it is possible that it is a cross between *P*. *illinoensis* and a linear-leaved species”. He clearly considered this view as tentative and appealed that “further collections and perhaps a study of the living plants will be needed to determine the exact nature of this plant”. Wilhelm & Mohlenbrock [59] conducted field surveys and discovered four morphologically uniform populations in the lower Blackwater River drainage, all in the vicinity of Milton, i.e. in the type area. Based on morphological comparison, they excluded the possible identity of *P*. *floridanus* with *P*. *natans* and *P*. *oakesianus*, which were unknown from the south-eastern United States, and with the Asian specimens identified as *P*. *tepperi*. They also rejected the hypothesis that it might be a hybrid and concluded that “observations in the field suggest strongly that these specimens represent a valid species”. Consequently, Wiegleb [29] listed *P*. *floridanus* among species of the *P*. *natans* group. In their worldwide account, Wiegleb & Kaplan [8] commented it as a unique plant, morphologically similar to a juvenile *P*. *natans*, but completely different in its stem anatomy, and brought back the hypothesis of hybrid origin. In their revision for the Flora of North America, Haynes & Hellquist [22] stated that the taxon persists and has a vegetative morphology unlike any other pondweed, and preferred to recognize it at specific level until a better understanding of the taxon would be developed. Lindqvist et al. [33] included *P*. *floridanus* in phylogenetic analyses and resolved it as a sister species of *P*. *oakesianus* based on the non-transcribed spacer of the nuclear ribosomal 5S region (5S-NTS) while two combined chloroplast intergenic spacers (*psb*A-*trn*H and *trn*L-*trn*F) grouped it with *P*. *oakesianus*, *P*. *nodosus*, *P*. *amplifolius* and *P*. *pulcher* that all appeared to have identical sequences with these markers. In a recent study dealing with typification of selected names of North American Potamogetonaceae, Kaplan & Reveal [60] commented *P*. *floridanus* as a taxonomically unclear taxon, probably of hybrid origin, and noted that all previous attempts to elucidate its exact identity have failed.

Nowadays, *P*. *floridanus* is considered as endemic to the lower Blackwater River drainage in Santa Rosa County, Florida [22, 59]. Because of its rarity, it was ranked as an endangered species in state [61–62], federal [63] and global IUCN [64] lists of endangered and threatened plants.

The review provided above shows that the identity of *P*. *floridanus* is still inconsistent and contradictory. Consequently, we decided to re-examine its status and taxonomic position using several molecular markers along with traditional tools. The aims of this study are: (1) to re-assess the identity of *P*. *floridanus* by testing its potential hybrid origin and other hypotheses with appropriate molecular markers, and (2) to check if morphology and stem anatomy are consistent with the molecular identification.

## Materials and methods

### Plant material

Field survey was conducted in the type area of *P*. *floridanus* in the lower Blackwater River drainage at Milton in 2012. A morphologically uniform colony of plants, which perfectly matched those from the type collection of *P*. *floridanus*, was sampled in the Pond Creek in Milton, which is a western tributary to the Blackwater River and one of the sites discussed by Wilhelm & Mohlenbrock [59]. Associated species included *P*. *diversifolius* and *Nymphoides aquatica* (Menyanthaceae).

In addition, we searched for specimens of *P*. *floridanus* in herbaria, examined all available specimens (or their photographs) including its holotype and isotype at NY, and more recent collections preserved at FLAS, GH, NASC, PRA and USF.

For molecular analyses, in addition to the fresh sample of *P*. *floridanus*, all species that were suggested as taxonomically identical, related or potentially involved in hybrid origin by morphology or molecular markers were included, namely *P*. *amplifolius*, *P*. *illinoensis*, *P*. *natans*, *P*. *nodosus*, *P*. *oakesianus*, *P*. *pulcher*, ‘*P*. *tepperi*’ (as delimited in [21]), three linear-leaved species (*P*. *berchtoldii*, *P*. *foliosus* and *P*. *pusillus*) and, additionally, *P*. *diversifolius* as a geographically co-occurring, but more distantly related species. As some of the listed North American species do not occur in Florida (see [22]), plant material was sampled in other parts of the country. The permission for sampling was granted by the United States Department of Agriculture. The specimens that had been previously used under the name ‘*P*. *tepperi*’ for comparison with *P*. *floridanus* actually belong to *P*. *distinctus* [8, 30]. Accessions of ‘*P*. *tepperi*’ from Australia were therefore complemented by samples of *P*. *distinctus* from Asia. The majority of the listed species are tetraploids whereas *P*. *berchtoldii*, *P*. *diversifolius*, *P*. *foliosus* and *P*. *pusillus* are diploids and *P*. *illinoensis* is octoploid [15]. Previous studies showed that due to founder effect and prevailing clonal growth, genetic variation is low or absent within populations of *Potamogeton* species [10, 14, 65–66], and entirely absent in sterile colonies of *Potamogeton* hybrids [67–71]. Also intraspecific genetic variation in *Potamogeton* is generally very low or absent [34, 45, 65], even at continental scales ([40, 42], and Fehrer & Kaplan, unpublished data). We therefore chose from our molecular database of Potamogetonaceae based on broad, worldwide sampling (including 3–32 populations of each above-mentioned species depending on rarity, 15 on average) single representative sequences of North American origin (except for the Asian / Australian taxa) per species and molecular marker except for *P*. *diversifolius* from Florida, of which we included two accessions. A list of samples and voucher information is provided in Table 1.

**Table 1:** *Potamogeton* samples used for molecular analyses.

Taxon	Identifier	Origin & voucher	GenBank accession numbers		
			ITS	*trn*T-*trn*L	5S-NTS
*P*. *diversifolius*	1770 (1)	USA: Texas, Jeff Davis Co., Davis Mountains, Cherry Canyon, Cherry Creek, 30°51'10"N, 104°03'28"W, coll. *C*. *B*. *Hellquist*, cult. & coll. *Z*. *Kaplan 1770* (PRA)	FJ151205	KY695277	KY695291
	1849 (2)	USA: Louisiana, Homer, Lake Claiborne, 32°47'55"N, 93°00'08"W, coll. *R*. *R*. *Haynes 10507* (PRA)	KY695267	KY695278	KY695292
*P*. *foliosus*	1608	USA: Vermont, Washington Co., Calais, Bliss Pond, 44°21'04"N, 72°30'05"W, cult. & coll. *Z*. *Kaplan 1608* (PRA)	KF270907	KY695279	KF270955
*P*. *pusillus*	1712	USA: Maine, Aroostook Co., Mars Hill, pond on Prestile Stream, 46°31'06"N, 67°52'01"W, coll. *Z*. *Kaplan & C*. *B*. *Hellquist 05/437* (PRA)	KF270914	KY695280	KF270989
*P*. *berchtoldii*	1641	USA: Connecticut, New London Co., Voluntown, Beachdale Pond, 41°35'05"N, 71°51'19"W, cult. & coll. *Z*. *Kaplan 1641* (PRA)	KY695268	KY695281	KF270938
*P*. *illinoensis*	1983	USA: Massachusetts, Berkshire Co., Adams, grown in pond, 42°36'17" N, 73°08'33" W, coll. *C*. *B*. *Hellquist 17102* (PRA)	KY695269	KY695282	KY695293
*P*. *nodosus*	2284	USA: Texas, Jeff Davis Co., Madera Canyon Road, Madera Creek, 30°39'34"N, 104°09'47"W, coll. *C*. *B*. *Hellquist 17191* (PRA)	KY695270	KY695283	KY695294
*‘P*. *tepperi’*	2364	Australia: Queensland, Charters Towers, Toomba Station, 19°59'18"S, 145°33'51"E, coll. *C*. *B*. *Hellquist 17218 & A*. *Leu* (PRA)	KY695271	KY695284	KY695295
*P*. *distinctus*	2675	India: Kashmir, distr. Badgam, Nilnag Lake, 33°51'20"N, 74°41'25"E, coll. *A*. *H*. *Ganie 1017* (PRA)	KY695272	KY695285	KF270952
*P*. *natans*	1756	USA: Massachusetts, Berkshire Co., Hancock, pond on Kinderhook Creek, 42°34'40"N, 73°17'51"W, coll. *Z*. *Kaplan & C*. *B*. *Hellquist 05/342* (PRA)	FJ151209	KY695286	KY695296
*P*. *oakesianus*	1628	USA: Massachusetts, Berkshire Co., Savoy, Bog Pond, 42°38'26"N, 73°01'59"W, coll. *Z*. *Kaplan & C*. *B*. *Hellquist 05/364* (PRA)	FJ151212	KY695287	KF270975
*P*. ×*floridanus*	2536	USA: Florida, Santa Rosa Co., Milton, Pond Creek, 30°36'25"N, 87°03'42"W, coll. *C*. *B*. *Hellquist 17239* (PRA)	KY695273 KY695274	KY695288	KY695297
*P*. *pulcher*	1681	USA: Massachusetts, Franklin Co., Orange, Lake Rohunta, 42°33'47"N, 72°16'23"W, coll. *Z*. *Kaplan & C*. *B*. *Hellquist 05/405* (PRA)	KY695275	KY695289	KF270987
*P*. *amplifolius*	2642	USA: New Hampshire, Carroll Co., Freedom, Upper Danforth Pond, 43°49'34"N, 71°06'14"W, coll. *C*. *E*. *Hellquist 243–12 & C*. *B*. *Hellquist* (PRA)	KY695276	KY695290	KY695298

### Molecular procedures

DNA was isolated using a sorbitol extraction method [72]. Three molecular markers were used to identify the origin of *P*. *floridanus*: two highly variable nuclear markers with biparental inheritance whose sequences allow species-level resolution and may show contributions of different parents in case of hybrid origin, and one chloroplast intergenic spacer, also allowing species-level resolution, as a uniparentally inherited marker to determine the direction of the cross in case of hybrid origin. In particular, the internal transcribed spacer (ITS) of nuclear ribosomal DNA is the most widely used marker to study close species relationships and hybridization in plants [73] and was already used successfully for the identification of many hybrids of *Potamogeton* (e.g., [40–41, 47, 74–75]). ITS sequences of *Potamogeton* species are well homogenized (uniform), even in polyploids, indicating diploidization of the genome [15, 47], and are therefore suitable for phylogenetic analysis and hybrid detection also at higher ploidy level. The even more variable 5S non-transcribed spacer (5S-NTS) was already applied for Potamogetonaceae and other families of aquatic plants [33]. This multi-copy marker was shown to be less well homogenized, even in diploids, but the polymorphisms show species-specific patterns and character additivity in *Potamogeton* hybrids, allowing the determination of parental species [45]. Two chloroplast markers used by Lindqvist et al. [33] were not sufficiently variable to distinguish between all species of interest, therefore the *trn*T-*trn*L chloroplast intergenic spacer employed by Iida et al. [32] for species-level resolution of Japanese *Potamogeton* was used. Chloroplast DNA is inherited maternally in *Potamogeton* [74].

The ITS region was amplified and sequenced as described in Kaplan & Fehrer [76], the procedure for 5S-NTS follows Kaplan et al. [15]. The *trn*T-*trn*L chloroplast intergenic spacer was amplified in a nested PCR approach because the yield of the products was usually too low for sequencing. The first PCR reaction was done in reaction volumes of 25 μl and contained 2.5 μl of Mg^2+^-free PCR buffer, 2 mM MgCl_2_, 0.2 mM of dNTP, 1 U of *Taq* DNA polymerase (Fermentas / Thermo Fisher Scientific, Vilnius, Lithuania), approximately 10 ng of DNA and 0.4 mM of primer 1 and primer 2 [32]. Temperature conditions were 95°C for 5 min for predenaturation, followed by 32 cycles of 95°C for 20 s, 62°C for 40 s, and 72°C for 1 min, and a final extension at 72°C for 10 min. PCR products were checked on a 1% agarose gel and diluted 1:10 when necessary; 1 μl of diluted or undiluted product served as a template in a second PCR with primer 3 and primer 4 [32] under the same reaction and cycling conditions. PCR products were purified using the QIAquick PCR purification kit (Qiagen, Hilden, Germany) and sequenced at GATC Biotech (Cologne, Germany) with primer 3, in case of difficult reads additionally with primer 4.

Direct sequences of the ITS region of *P*. *floridanus* showed traces of a second ribotype (S1 Fig). In order to retrieve this variant, products of three separate PCR reactions (triplicates) were pooled and cloned as described in Fehrer et al. [77]. Clones were subjected to discriminating restriction fragment length polymorphism (RFLP) analysis to pre-screen for the underrepresented sequence. Restriction enzyme *Pae*I (recognition sequence GCATGC) was chosen to distinguish the major sequence by a restriction site which was lost in the underrepresented variant (S1 Fig). Restriction digests were done using 10 μl of PCR product (corresponding to 10–50 ng of amplified DNA), 5 U of enzyme and 1/10 of buffer Blue 1x (Fermentas / Thermo Fisher Scientific) at 37°C for 16 h, followed by 65°C of inactivation for 20 min. Resulting fragments were separated on a 1% agarose gel stained with ethidium bromide. A single clone of the required pattern (uncut PCR product) was obtained and sequenced with the forward PCR primer.

Sequences of all datasets were submitted to the GenBank database; accession numbers are included in Table 1.

### Molecular data analyses

Sequence electropherograms were edited manually using Chromas v.1.45 (Technelysium Pty Ltd., Australia) and aligned by hand in Bioedit v.7.0.9.0 [78]; alignments were unambiguous for all markers. For the 5S-NTS dataset, available sequences of the same species from the study of Lindqvist et al. [33] were retrieved from GenBank and added to the alignment. Indel coding for all datasets was performed with FastGap v.1.2 [79] based on the simple method of Simmons & Ochoterena [80]. The individual datasets were subjected to maximum parsimony (MP), maximum likelihood (ML) and Bayesian analysis in order to show the position of *P*. *floridanus* in phylogenetic trees. MP and ML analyses were performed with PAUP* v.4.0b10 [81]; Bayesian inference was done with MrBayes v.3.1.2 [82]. The most divergent species, *P*. *diversifolius*, was used as an outgroup. MP and ML analyses were done as heuristic searches with 100 random addition sequence replicates and TBR branch swapping, saving no more than 100 trees with length greater than or equal to 1 per replicate. Bootstrapping was done using the same settings and 1000 replicates, but without branch swapping. For the larger 5S-NTS dataset, ML analysis was omitted. For ML and Bayesian analyses, the model best fitting the presumed molecular evolution of the respective datasets was determined using Modeltest v.3.5 [83]. In hierarchical Likelihood Ratio Tests, a K80+G model was found for both ITS and 5S-NTS, and a F81+G model for *trn*T-*trn*L. The respective likelihood settings were used for ML analyses. For Bayesian analyses, only the basic model parameters (gamma distribution for all datasets, two substitution rates for K80+G, and one substitution rate for F81+G) were set as priors. One million generations were run with a sample frequency of every 1000^th^ tree. Statistical parameters indicated that convergence was reached in all datasets. The first 25% of the trees were discarded as burn-in, and the rest of the trees were summarized.

5S-NTS sequences usually contained high numbers of intra-individual polymorphisms (sometimes at more than 10% of the positions), even in non-hybrid species (S2 Fig, S1 Table). They were distinguished from noise if they were visible in forward and reverse sequence reads. In some cases, direct sequences were very poorly readable because of high numbers of polymorphisms or indel mutations and had to be cloned to obtain reliable sequence reads and to identify the exact position and length of indels. In order not to introduce too much noise in phylogenetic analyses, ‘major’ sequences were additionally generated: Small additional peaks that did not constitute more than about 30% of the total signal in both reading directions and that did not show indications of character additivity with other species were ignored.

### Investigation of morphology and stem anatomy

A new and comprehensive morphological assessment of *P*. *floridanus* was carried out, based on all available herbarium specimens and on fresh material recently collected in the type area. All characters traditionally used in taxonomy of *Potamogeton* species (e.g. [8, 58]) were scored.

Stem anatomy is an important source of additional characters for resolving taxonomic difficulties in *Potamogeton*, identification of specimens lacking essential morphological features and for detection of hybrids between species with different anatomical types (e.g., [25, 28, 30–31, 57–58, 84–87]). We therefore examined the stem anatomy of a recently collected herbarium specimen (Hellquist 17239, PRA). We did not dissect the stems of the original type collections in order not to damage the specimens, but observations made and indicated by Ogden on annotation labels were considered.

Short pieces of stem were cut from the middle of the internode of the main stem, soaked for a few days in a solution of equal parts of water, ethanol and glycerol. Approximately 0.05 mm thick slices from the stem fragments were cut transversally with a razor blade under a stereomicroscope and then stained in aqueous solution of toluidine blue for 1–3 minutes. Stained tissue was subsequently washed in distilled water. Stem anatomy was investigated using a transmission light microscope at magnifications between 50× (general anatomical pattern) and 400× (details).

The anatomical terminology used follows Wiegleb [88] and Wiegleb & Kaplan [8]. Line drawings of important anatomical structures (interlacunar and subepidermal bundles, pseudohypodermis) are given by Ogden [58] and Symoens et al. [89] and coloured photographs of these by Kaplan [90].

## Results

### Molecular markers indicate *P*. *floridanus* is a hybrid between *P*. *pulcher* and *P*. *oakesianus*

**Fig 1:**
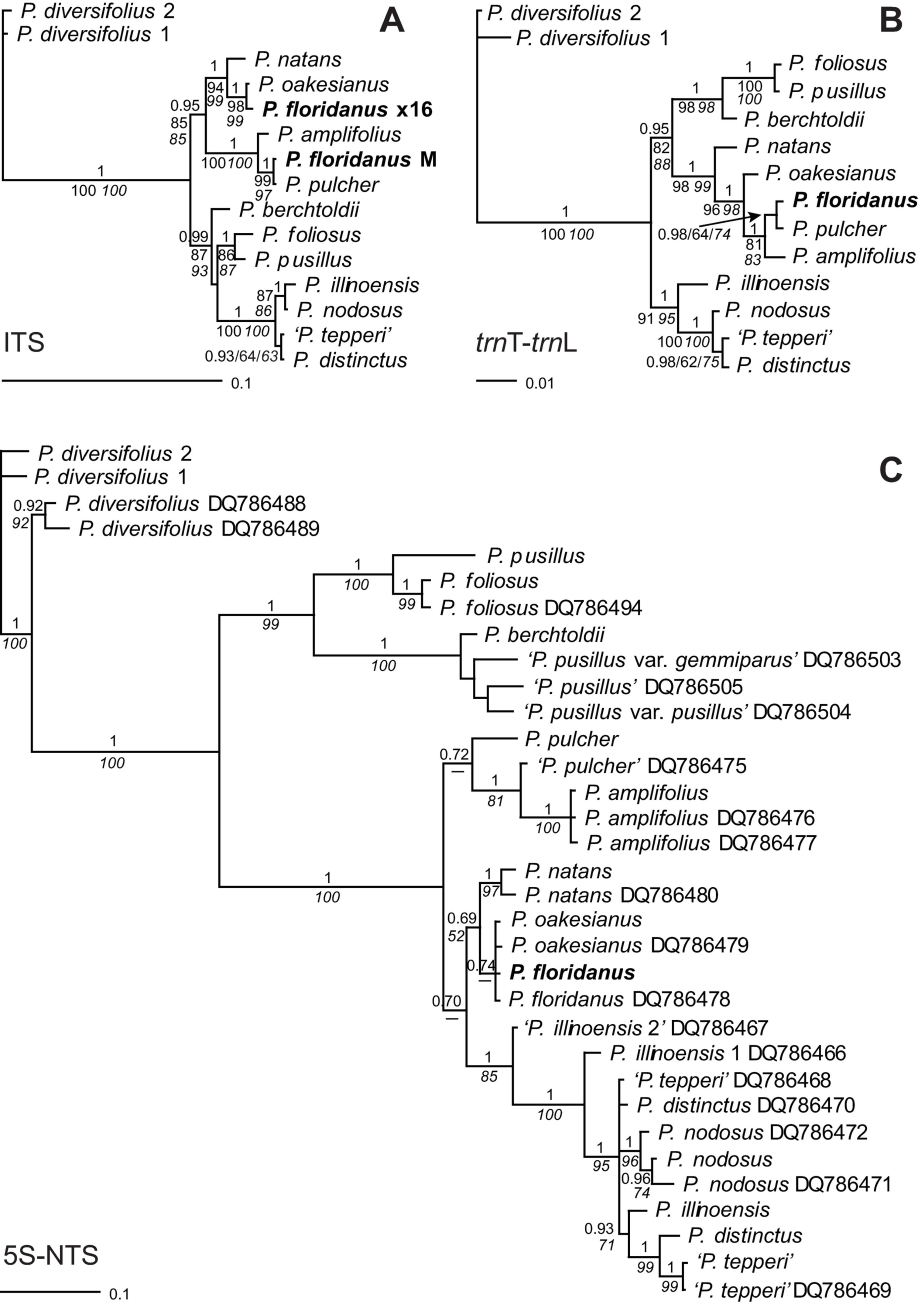
Placement of *P*. *floridanus* in phylogenetic trees. Bayesian consensus trees are shown with posterior probabilities above and bootstrap support of ML and MP analyses below branches (MP support in italics). **A**: The major ITS sequence of *P*. *floridanus* (M) corresponds to that of *P*. *pulcher*; a cloned sequence of the underrepresented ribotype of *P*. *floridanus* (x16) groups with *P*. *oakesianus*. **B**: The *P*. *floridanus* chloroplast haplotype corresponds to that of *P*. *pulcher* indicating the maternal origin of the hybrid. **C**: 5S-NTS sequences of the same species from Lindqvist et al. [33] were included; they are indicated by GenBank accession numbers after species names. As in that study, *P*. *floridanus* groups with *P*. *oakesianus*, albeit without significant support. This placement is largely an artifact due to sequence polymorphisms (see text).

Direct sequencing of the ITS region revealed that *P*. *floridanus* had a ribotype corresponding to that of *P*. *pulcher* (Fig 1A), which was not evident from morphology. Also, the chloroplast *trn*T-*trn*L sequence was identical with that species (Fig 1B), unequivocally indicating *P*. *pulcher* also as the maternal parent of *P*. *floridanus*. However, identity of the two taxa could be excluded based on morphology. Closer inspection of the ITS sequence electropherogram showed some very small additional peaks that suggested a contribution from *P*. *oakesianus* according to some readable diagnostic single nucleotide polymorphisms (SNPs) and two diagnostic 1 bp-indels that caused a shift in ITS1 and a backshift in ITS2; the amount of these ITS copies constituted ca 2–5% of the total signal (S1 Fig). Out of 39 cloned sequences, only a single clone of the ribotype representing the second parent (corresponding to ca 2.5% of the screened clones) was found. Its sequence was identical to that of *P*. *oakesianus* (Fig 1A) except for one unique substitution that most probably represents a PCR artifact (polymerase error).

**Fig 2:**
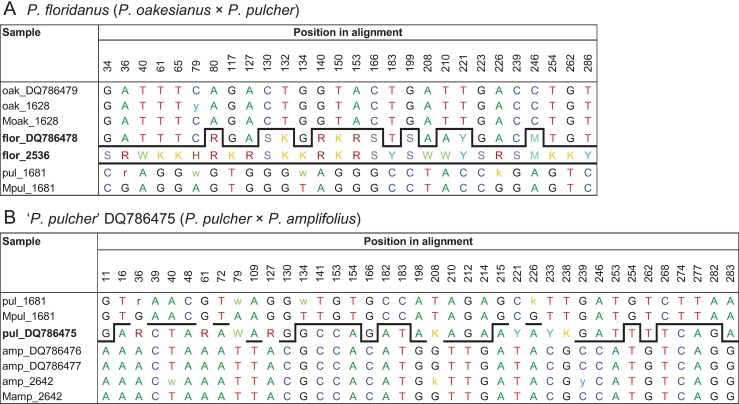
Character additivity and major 5S-NTS sequences of hybrid accessions. oak = *P*. *oakesianus*, flor = *P*. *floridanus*, pul = *P*. *pulcher*, amp = *P*. *amplifolius*; hybrid accessions are in bold. Only positions at which both parents differ are shown; sites containing intra-individual polymorphisms in only one sample or species are omitted. Numbers after species abbreviations are accession identifiers, GenBank accession numbers indicate sequences from Lindqvist et al. [33]. ‘M’ before a sample name indicates the major sequence corresponding to much higher peaks in the electropherogram (S2 Fig) in both reading directions at double peak positions. Letters other than G, A, T and C are IUPAC ambiguity codes: S = C or G, R = A or G, W = T or A, K = T or G, Y = C or T, M = C or A, and H = C or A or T. Colours and bold lines are for easier orientation.

As the ITS ribotype of *P*. *oakesianus* was present in very low amount, we sequenced a further nuclear marker to confirm the contribution of that species to *P*. *floridanus*. A phylogeny based on 5S-NTS had previously indicated a sister relationship between these taxa [33]. For comparison with our accessions, sequences from that study were retrieved from GenBank and included in phylogenetic analyses. Generally, accessions of the same species from both studies grouped together (Fig 1C). As already observed by Lindqvist et al. [33], sequences of *P*. *floridanus* and *P*. *oakesianus* clustered together in the 5S-NTS trees. However, this clade was neither supported nor significantly separated from our accession of *P*. *pulcher* as all branches in between those three taxa were unsupported. This was most likely caused by polymorphisms in 5S-NTS sequences of *P*. *floridanus* that were additive for *P*. *pulcher* and *P*. *oakesianus* (Fig 2A) unequivocally demonstrating both species as parents of *P*. *floridanus*.

The involvement of the other species previously considered to be identical or related to *P*. *floridanus* can be excluded because their sequences differ from that of *P*. *floridanus* in all three markers. Particularly the superficially similar Australian (‘*P*. *tepperi*’) and Asian plants (*P*. *distinctus*) are considerably distant both in terms of the number of substitutions and in their placements in the phylogenetic trees.

### Morphological assessment

*Potamogeton floridanus* resembles *P*. *oakesianus* with which it shares its general appearance and basic superficial features such as shape and size of leaves, and may therefore be almost indistinguishable morphologically. Submerged leaves are reduced to linear phyllodes, which are 0.5–1.5 mm wide and have only one vein. Petiolate floating leaves are present on adult plants that reach the water surface. Their lamina is narrowly oblong to oblong, 35–83 mm long, 4–22 mm wide, coriaceous, 3.5–16 times longer than wide, 7–13-veined, acute at the apex, cuneate at the base. The petiole is 48–295 mm long, 1.5–5.1 times as long as the lamina, mostly without a discoloured section at the junction with the lamina but sometimes with indistinct traces of the discoloured section. Some of the uppermost submerged leaves are transitional in shape between submerged and floating leaves, which is typical of hybrids between species with laminar submerged leaves (such as *P*. *illinoensis*, *P*. *nodosus* and *P*. *pulcher*) and those with phyllodial submerged leaves (*P*. *natans* and *P*. *oakesianus*; see [12, 71]). Stipules are axillary, convolute, 18–63 mm long. Peduncles are 49–92 mm long, bearing a cylindrical spike, which is 9–11 mm long. Fruits have never been observed and are presumably not produced, as in the great majority of *Potamogeton* hybrids, which are consistently sterile (e.g. [8–9, 11, 16, 47, 57, 91–93]).

### Investigation of stem anatomy

Stem anatomy of the recently collected specimen (Hellquist 17239, PRA) shows the stele to be of proto type and the endodermis of (O–)U-type. No interlacunar or subepidermal bundles were observed in the cortex, thus they were either absent or too weakly developed. Pseudohypodermis was only represented as a very short section at the periphery of one stem sample, otherwise it was absent. Annotation labels attached by Ogden to the holotype and isotype of *P*. *floridanus* indicate a very similar anatomy: stele of proto type, endodermis of U-type, interlacunar bundles present but weakly developed, subepidermal bundles absent or only weakly developed and pseudohypodermis absent. This anatomical pattern is fully consistent with the results of molecular analysis as it shows a combination of characters of the parental species: The hybrid shares its stele type with *P*. *pulcher* whereas the thickening of the endodermis cell is like that in *P*. *oakesianus*. In most other anatomical characters, *P*. *floridanus* occupies an intermediate position between its parents (Table 2).

**Table 2:** Comparison of the most relevant anatomical characters of *Potamogeton* ×*floridanus* and its parental species. Anatomical patterns of *P*. *oakesianus* and *P*. *pulcher* are based on observations of numerous samples as summarized in Ogden [58] and Wiegleb & Kaplan [8].

Taxon (specimen)	Type of stele	Shape of thickening in the endodermal cells	Presence of interlacunar bundles	Presence of subepidermal bundles	Presence of pseudohypodermis
*P*. *oakesianus*	trio type	U-type	present in 1–2 circles	present	absent
*P*. *pulcher*	proto type	O-type	absent	absent	present in 1 layer
*P*. ×*floridanus* (May 1886, *A*. *H*. *Curtiss s*. *n*., NY, holotype)	proto type	U-type	present	absent or present but weakly developed	absent
*P*. ×*floridanus* (May 1886, *A*. *H*. *Curtiss s*. *n*., NY, isotype)	proto type	U-type	present but weakly developed	present	absent
*P*. ×*floridanus* (*Hellquist 17239*, PRA)	proto type	(O–)U-type	not seen (absent or too weakly developed)	absent	very short sections of 1 layer present, otherwise absent

## Discussion

### Identity and origin of *P*. ×*floridanus*

Molecular analysis revealed that *P*. ×*floridanus*, until now considered a distinct, endemic and endangered species, is actually a hybrid *P*. *pulcher* × *P*. *oakesianus*. Detailed morphological and anatomical investigations were in agreement with this identification. Morphologically, the hybrid is most similar to *P*. *oakesianus*, whilst the dominant ITS sequence and the chloroplast *trn*T-*trn*L sequence are those of *P*. *pulcher*. The genetic involvement of *P*. *oakesianus* was shown by a single cloned ITS sequence. In addition, 5S-NTS sequences showed complete character additivity at all positions that differed between the parental species. The stem anatomy also combines the characters of *P*. *oakesianus* and *P*. *pulcher*.

The discovered identity of *P*. ×*floridanus* is rather surprising because one of its parental species, *P*. *oakesianus*, currently does not occur in Florida, and its nearest sites are as distant as in Virginia [22] approximately 1,100 km away. However, previous studies conducted in Europe showed that *Potamogeton* hybrids can persist vegetatively in the absence of the parental species, presumably being remnants after one or both parents disappeared (e.g. [11–12, 41, 75–76, 94]). For example, Kaplan & Fehrer [40] identified a sterile clone of *P*. *gramineus* × *P*. *nodosus* persisting for a long time in Sweden although *P*. *nodosus* currently does not occur in the whole of Scandinavia because it is adapted to warmer climate.

The occurrence of *P*. ×*floridanus* in Florida can be explained by two hypotheses. The first involves a hybridization event that may have occurred in the area of the sympatry of the parental species in the north-eastern USA and a subsequent long-distance transport of the hybrid seed to the site in Florida. Although migrating birds are known as a means of transport of plant propagules (e.g. [95–96]), coincidence of several factors is necessary if the seed dispersal, seed germination and new population establishment should be successful [97]. Therefore, ornithochory is much more likely to contribute to the dispersal of those aquatic plant species that are frequent in the landscape, form relatively large biomass with numerous seeds and possess a broad ecological range [94, 98–102]. In case of *P*. ×*floridanus* it seems rather unlikely that a hybrid seed of this unique combination would establish a hybrid colony only in such a distant and ecologically different area. A second hypothesis is therefore more probable: *P*. ×*floridanus* represents a hybrid clone persisting from the past when both parental species still co-occurred in Florida. Hybridization has recently been suggested as a mechanism of survival of endemic species in times of climate change [103]. At the time of the last glaciation, *P*. *oakesianus* may have occupied a much more southerly range than today and together with *P*. *pulcher* may have given rise to the local hybrid population. One of the parental species, adapted to different ecological conditions, later disappeared due to the climatic and vegetation changes in the following early postglacial period. In contrast, the hybrid clone may be fitter than the parents due to heterosis resulting from mixing the genetic contributions of its parents [104] and stabilized by vegetative growth. The hypothesis of the long-term persistence of *P*. ×*floridanus* is supported by herbarium specimens, which document its existence at least since the 19th century.

### Discrepancies in 5S-NTS sequence data in different studies

Our accession of *P*. ×*floridanus* showed complete character additivity in the 5S-NTS region at all 28 diagnostic positions. In contrast, Lindqvist et al. [33] indicated only 10 of these positions as polymorphic in *P*. *floridanus* DQ786478 whereas 17 positions showed the *P*. *oakesianus*-specific character states, and at one position (210), the character state corresponded to *P*. *pulcher* (Fig 2A). The resulting sequence appears to have been somewhat artificially ‘corrected’ towards the presumably slightly dominating peaks of *P*. *oakesianus*, either by hand or, more probably, by the automatic base caller. Consequently, *P*. *floridanus* appeared to be a sister species of *P*. *oakesianus* instead of being recognized as its hybrid. Thus, the position of *P*. *floridanus* in the phylogenetic tree (Fig 1C) represents an artifact caused by polymorphic characters that are actually additive for different species.

According to ITS and *trn*T-*trn*L trees (Fig 1A and 1B) and character additivity in 5S-NTS (Fig 2A), one parent of *P*. *floridanus* is *P*. *pulcher*. In the 5S-NTS tree, the position of *P*. *pulcher* DQ786475 differs from that of our accession (Fig 1C). Closer inspection of character states showed that this sample actually represents a previously unrecognized hybrid between *P*. *pulcher* and *P*. *amplifolius* (Fig 2B). Out of 39 positions that differ between these species, DQ786475 contains 8 positions with additive character states of both species; at 13 positions, the character states are specific for *P*. *pulcher*, but at 18 sites, they correspond to *P*. *amplifolius*. Thus, also this sequence read appears to have been artificially ‘resolved’ towards the respective higher peaks. The result is a chimera of parental character states. The ribotypes of both parents have probably been present in approximately equal amounts in the respective sample, and reaction-typical variation of relative peak heights in sequence reads were overinterpreted or resolved automatically. This is in keeping with the position of the chimeric sequence between *P*. *pulcher* and *P*. *amplifolius* in Fig 1C. Thus, no sample of pure *P*. *pulcher* was actually included in the study of Lindqvist et al. [33]. This may also be a reason why these authors could not observe the quite obvious character additivity of *P*. *pulcher* and *P*. *oakesianus* in their accession of *P*. *floridanus*.

Similarly, ‘*P*. *illinoensis* 2’ DQ786467 clusters in a relatively basal position in the 5S-NTS tree, relatively far away from its conspecific ‘*P*. *illinoensis* 1’ DQ786466. The latter clusters nearer our sample of *P*. *illinoensis*, basal of a clade comprising also *P*. *nodosus*, ‘*P*. *tepperi*’ and *P*. *distinctus*. Although *P*. *illinoensis* 1 contains a much higher number of polymorphic sites than ‘*P*. *illinoensis* 2’ (S1 Table), the latter shows partial character additivity with samples not included in the present study (data not shown). In the 5S-NTS tree presented in Lindqvist et al. [33], this sample occurred in a basal and intermediate position between relatively divergent clades, a result which the authors could not interpret and simply stated that ‘more work is needed on the *P*. *illinoensis* group’. We consider it most likely that this sample also represents a misidentified hybrid involving *P*. *illinoensis* as one of its parents, but the exact identity of the second parent cannot be inferred with the available data in this case.

The slightly different positions of some ‘*P*. *tepperi*’ and *P*. *distinctus* samples (Fig 1C) may be due to intraspecific variation comparable to that in other widespread species. Three samples designated as ‘*P*. *pusillus*’ by Lindqvist et al. [33] actually correspond to *P*. *berchtoldii*, a species widely misunderstood in North America [34, 60]. No sample of true *P*. *pusillus*, which is closely related to *P*. *foliosus*, but genetically well distinguished from *P*. *berchtoldii* (see also [10, 15]) was included in Lindqvist et al. [33], but the discrepancy in this case was caused only by misidentified plant material and not an erroneous handling and interpretation of sequence data.

Given the generally high level of polymorphic sites in 5S-NTS sequences (S1 Table, S2 Fig) [45] and some of their most prominent patterns (Fig 2), the statement of Lindqvist et al. [33] that “only very few intraindividual polymorphic sites were found, and these seemed to appear in a random phylogenetic pattern” is obviously wrong.

### Failure of hybrid recognition: Reasons and consequences

Although botanists have attempted to resolve the taxonomic identity of the Florida pondweed for more than 130 years, they have been unsuccessful and have not come to satisfactory conclusions. This had several reasons. First, although some of the earlier researchers correctly suspected that *P*. ×*floridanus* may be a hybrid, they always considered only local diversity when looking for the potential parents, while the local diversity could not explain the observed morphological and anatomical patterns. Ogden [58] in his discussion on the identity of *P*. ×*floridanus* mentioned, among others, both *P*. *pulcher* (“The stelar pattern of the plant in question leads me to consider *P*. *pulcher* as a possible parent”) and *P*. *oakesianus* (“It is possible that the plant is a pronounced ecological form of *P*. *Oakesianus*”) but finally rejected both species as the parents. A second reason may be the rather uninformative morphology of *P*. ×*floridanus*. Although many hybrids are clearly intermediate between their parents and can be identified once one becomes familiar with the range of intraspecific variation (e.g. [9, 16, 57]), others can be revealed and correctly recognized only by molecular analysis [11, 40, 44]. *Potamogeton* ×*floridanus* falls in the latter group because the variation in its morphology overlaps that of *P*. *oakesianus* and both taxa are highly plastic. A third reason is that molecular methods, which appear to be necessary for resolving cases like this, became available only during the past decades, and our re-analyses show that they have been applied inadequately in a previous phylogenetic study involving *P*. *floridanus* [33].

Although molecular procedures are now well established and widely applied, molecular identification of hybrids is still not straightforward. Sequence data generation has become so easy and highly automized that almost every molecular lab in the world can produce raw data without problems. DNA barcoding initiatives are further pushing into the direction of easy-to-apply, standardized markers, but one-for-all solutions are not applicable in plants (reviewed by Li et al. [105]). The basic question remains whether barcodes are a part or a consequence of species definitions (for discussion, see Fehrer et al. [106]). The sole use of chloroplast DNA for molecular identification must fail for hybrids, because only one of the parents can be determined in principle. Given that hybridization in plants is the rule rather than the exception and that chloroplast capture can occur even between different genera [107], identifications based solely on chloroplast data must lead to erroneous conclusions in case of hybrid or allopolyploid origin. This is also the case here: chloroplast DNA would have identified *P*. *×floridanus* as *P*. *pulcher*, leaving the puzzle why it does not look like that species. Nuclear markers such as the ITS region have been sometimes used as an additional DNA barcode for plants [105] and also been recommended for Potamogetonaceae [108]. But as our example shows, extremely skewed ratios of parental ITS copies would, without careful manual inspection of sequence electropherograms, have led to the wrong conclusion that the hybrid is indeed identical with *P*. *pulcher*. (This does not, however, argue against the use of the ITS region for molecular identification of Potamogetonaceae in general, because typically this region allows to recognize at first glance whether the sequence of a particular sample belongs to a hybrid or not.) Only comparison of very small additional peaks and the position of diagnostic indels with our database of *Potamogeton* ITS sequences (Fehrer et al., in preparation) suggested an involvement of *P*. *oakesianus*, which was subsequently confirmed by a sophisticated cloning approach with pre-selection of the rare ITS type by RFLP screening. The 5S-NTS region is unsuitable in *Potamogeton* for DNA barcoding purposes despite the fact that it provides excellent species-level delimitation in most cases [15]. This is due to the extremely high level of polymorphisms that indicate rapid molecular evolution, but poor concerted evolution of this non-coding marker. The advantage of this feature is that hybrid signatures appear to prevail longer in the 5S-NTS region so that parental copies can still be traceable with this marker when they have already disappeared in the ITS region. Also in case of *P*. *×floridanus*, we found approximately equal ratios of 5S-NTS copies of both parents. Lindqvist et al. [33] who investigated the same taxon from the same site with the same marker failed, however, to correctly interpret the sequence data, also in two other cases mentioned above. The result is spurious conclusions about species identities, their phylogenetic relationships and intraspecific variation. The trend to increasingly rely on automized sequence reads and/or uncritical manual treatment (inappropriate ‘corrections’ towards the respective higher peaks), especially in critical plant groups whose taxonomy and morphological species circumscription is not yet sufficiently understood, shows the necessity to at first establish a reliable molecular identification system based on thorough taxonomic work before DNA barcoding can be applied reliably by non-experts.

Lindqvist et al. [33] have used herbarium material of the University of Alabama for their molecular phylogenetic study. Of this material, as far as it was compared here with our own samples, at least five accessions (three *P*. *pusillus*, *P*. *pulcher*, and one *P*. *illinoensis*) were wrongly determined, and two of these were apparently hybrids. This means, together with *P*. ×*floridanus*, at least three hybrids were used unassumingly for a phylogenetic study, resulting in placements of taxa that clustered either with one parent (*P*. ×*floridanus*) or ended up between parents or parental clades (*P*. *pulcher*, *P*. *illinoensis*), which are typical patterns resulting from the use of polymorphic sequences of hybrids in phylogenetic analyses [109–110]. 5S-NTS sequences of *Potamogeton* generally show a high level of polymorphisms within individuals that are more prominent in hybrids, but not restricted to them (S1 Table) [45]. This example highlights the fundamental importance of correct species identifications for any kind of studies on evolution, ecology or biodiversity. Obviously, a phylogeneticist, ecologist or geneticist who is not expecting to deal with hybrids can be easily mislead, which results in erroneous entries in public sequence databases by which further work is affected. This is particularly true for *Potamogeton*, a genus in which the existence of hybrids in North American floras has been doubted and therefore ignored for a long time [11]. This example highlights the importance of sound taxonomic work, especially in plant groups with highly reduced morphology and high phenotypic plasticity.

### Consequences for nature protection

In a changing world, we face a considerable loss of plant species diversity. We urgently need an appropriate biodiversity assessment [111] and accurate information on species delimitations, origins, distributions and threats. State-of-the-art taxonomic evaluation is an indispensable first step for understanding patterns of biological diversity and identification of conservation priorities and adequate conservation measures. A lack of taxonomic knowledge poses considerable problems for conservationists [112–113]. Taxonomic revisions, both as case studies and monographs, are the most important and often the only source of data on accurate assessment of biodiversity [114–115]. Comprehensive taxonomic studies combining molecular, cytogenetic, morphological and ecological approaches have resulted in remarkable discoveries even in well-known floras [116]. We therefore urge on modern taxonomic re-evaluation of poorly known distinct populations.

Modern conservation programs should include not only endangered species and habitats, but also consider evolutionary processes such as hybridization, segregation and natural selection, which generate taxonomic biodiversity [117]. Conservation guidelines for hybrids are essential in preserving biodiversity, but still remain to be established [118]. For example, the EU Water Framework Directive does not recognize hybrids as indicators for a good ecological state. A river hosting abundant stands of a correctly identified hybrid of *Ranunculus peltatus* (sampled by an experienced taxonomist) would be ‘empty’, while the same river recorded to host non-hybrid *R*. *peltatus* (as it would be probably incorrectly identified by an untrained field biologist) would have a ‘good quality’. However, from an ecological point of view both taxa have the same positive functions in the river ecosystem and occupy the same niche. Although our research identified *P*. ×*floridanus* as a sterile hybrid of two extant species and not as a “normal” fertile species, threatened populations of hybrids may still represent important components of biological diversity [119], and the ecological context is worth being considered for a balanced strategy of conservation efforts [120]. *Potamogeton* ×*floridanus* represents a unique element of aquatic biodiversity with a peculiar history of evolutionary and ecological processes involving hybridization and long-term survival of a sterile clone, and it is a dominant plant in local aquatic communities.

## Supporting information

S1 Fig Electropherograms of forward and reverse direct sequence reads of the ITS region of *P*. *floridanus*. (PDF)

S2 Fig Electropherograms of forward and reverse direct sequence reads of the 5S-NTS region of all samples. (PDF)

S1 Table Polymorphisms in direct and major 5S-NTS sequences. (PDF)
