# Supplementary material for: The endangered Florida pondweed (Potamogeton floridanus) is a hybrid: Why we need to understand biodiversity thoroughly
Source: PLoS One. 2018 Apr 2;13(4):e0195241. doi: 10.1371/journal.pone.0195241 (PMC5880373; doi:10.1371/journal.pone.0195241)

Supplementary Figure S1:

Electropherograms of forward and reverse direct sequence reads of the ITS region of *P. floridanus*

Asterisks indicate positions with low amounts of *P. oakesianus* characters (the major sequence corresponds to *P. pulcher*). Most are visible on both reads. Down arrows show two shifts caused by 1 bp-indels compensating each other. A discriminating *Pae*I restriction site (GCATGC) present only on the major sequence is shown by a box in the first row.

Po2536_ITSF

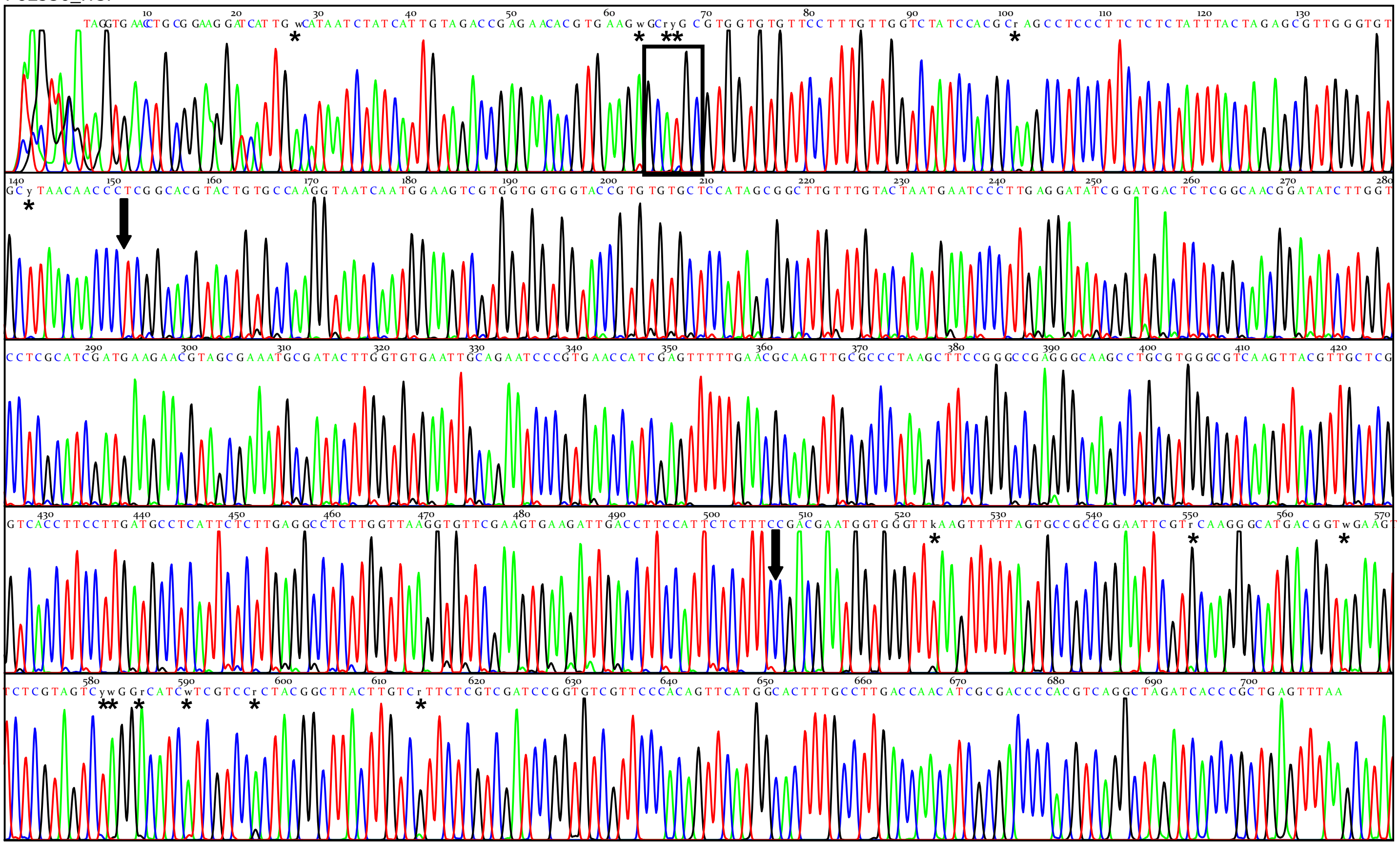

Po2536_ITS4 reverse complement

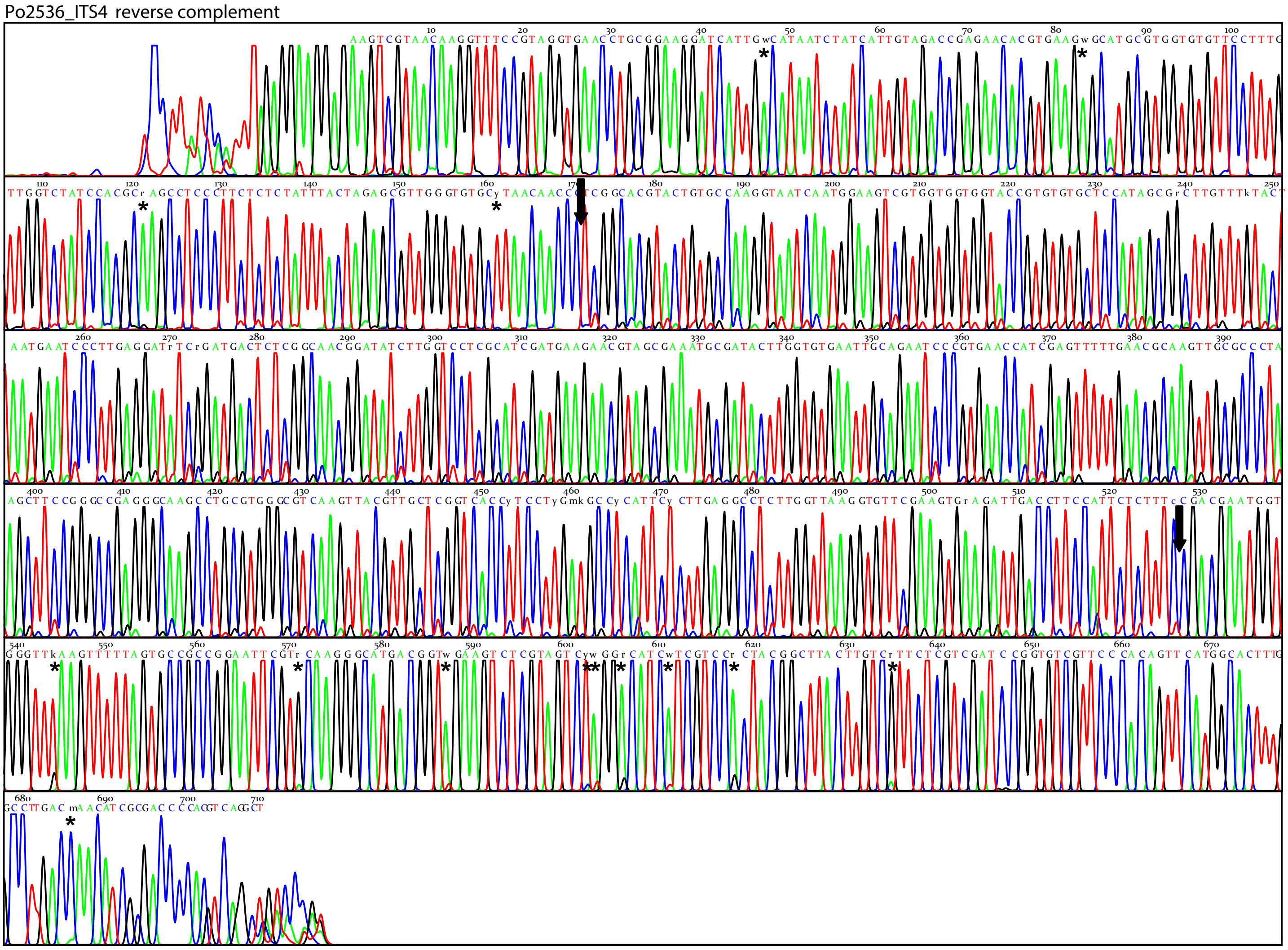

Supplement: S1 Fig — (PDF) [file pone.0195241.s001.pdf]
