# Supplementary material for: The endangered Florida pondweed (Potamogeton floridanus) is a hybrid: Why we need to understand biodiversity thoroughly
Source: PLoS One. 2018 Apr 2;13(4):e0195241. doi: 10.1371/journal.pone.0195241 (PMC5880373; doi:10.1371/journal.pone.0195241)

P. diversifolius 1770 5S-NTS forward

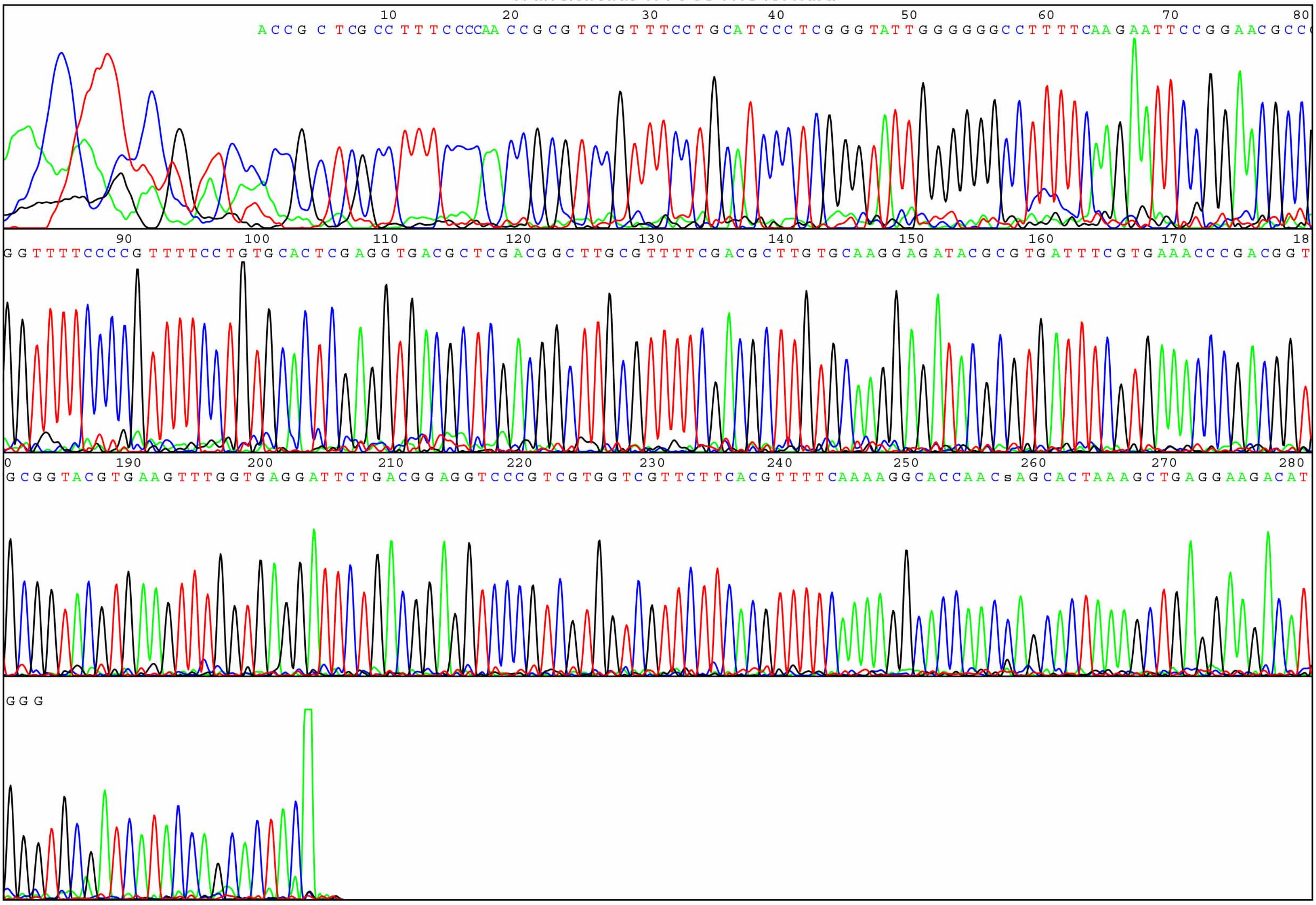

P. diversifolius 1770 5S-NTS reverse

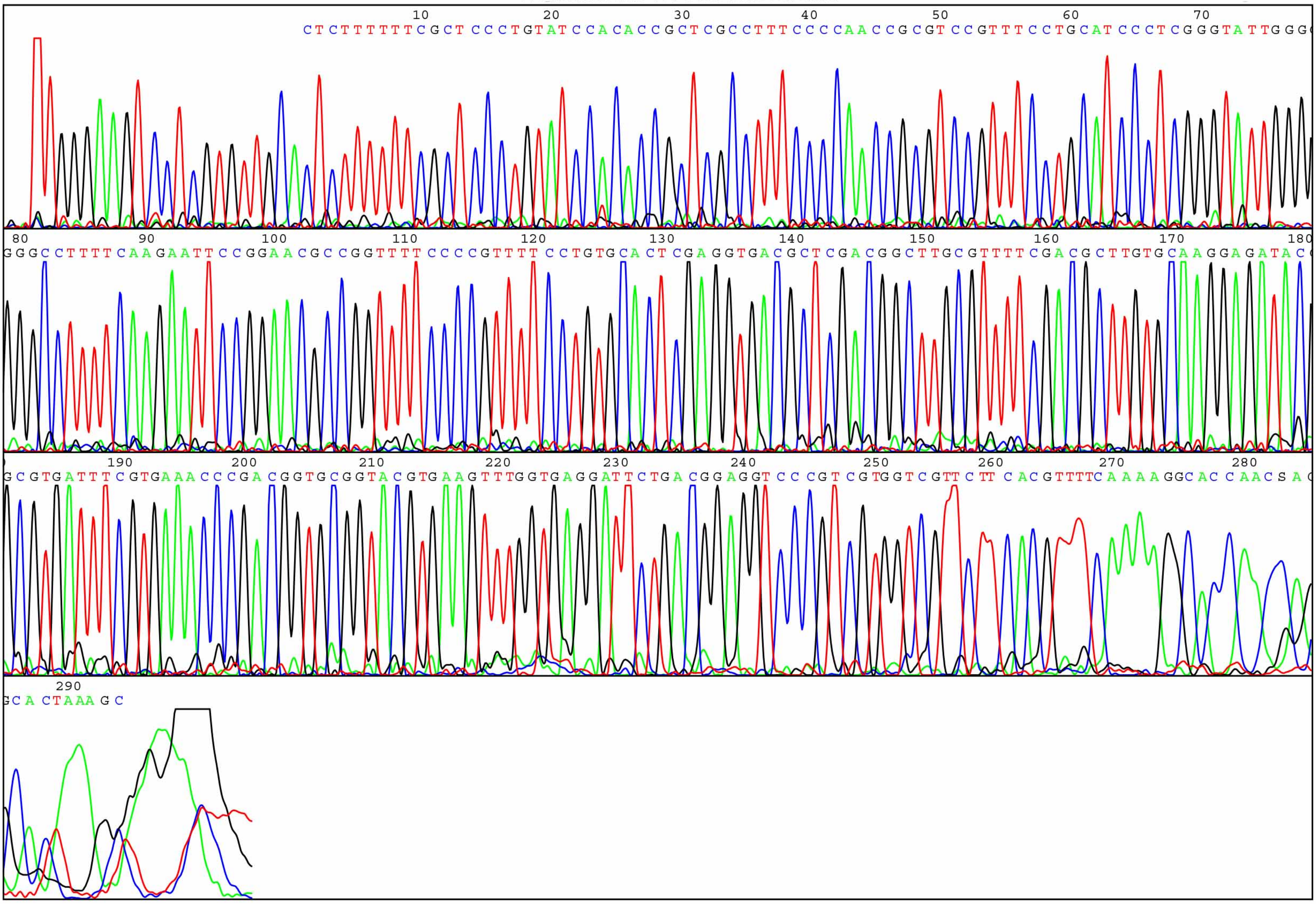

P. diversifolius 1849 5S-NTS forward

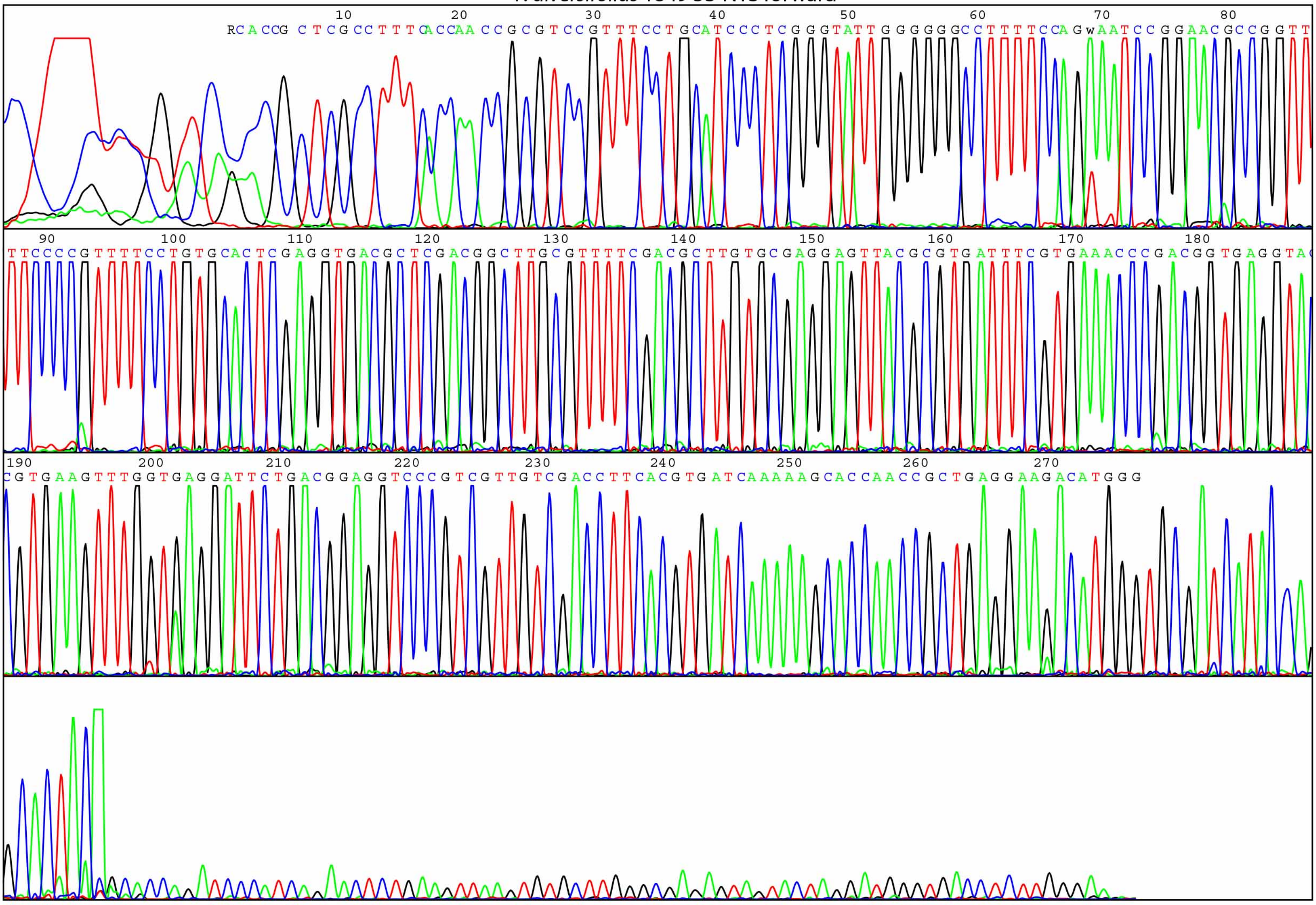

P. diversifolius 1849 5S-NTS reverse

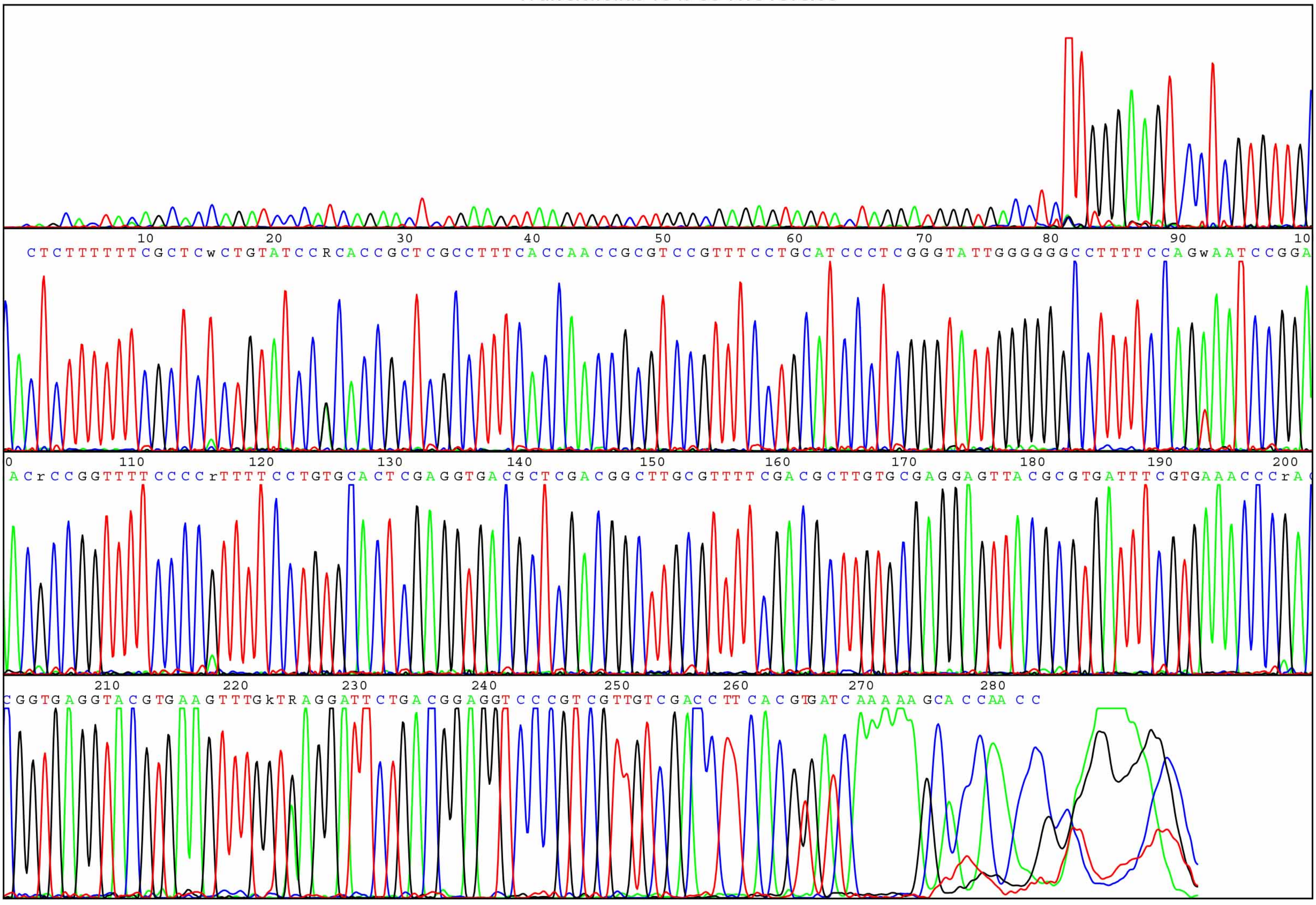

P. foliosus 1608 5S-NTS forward

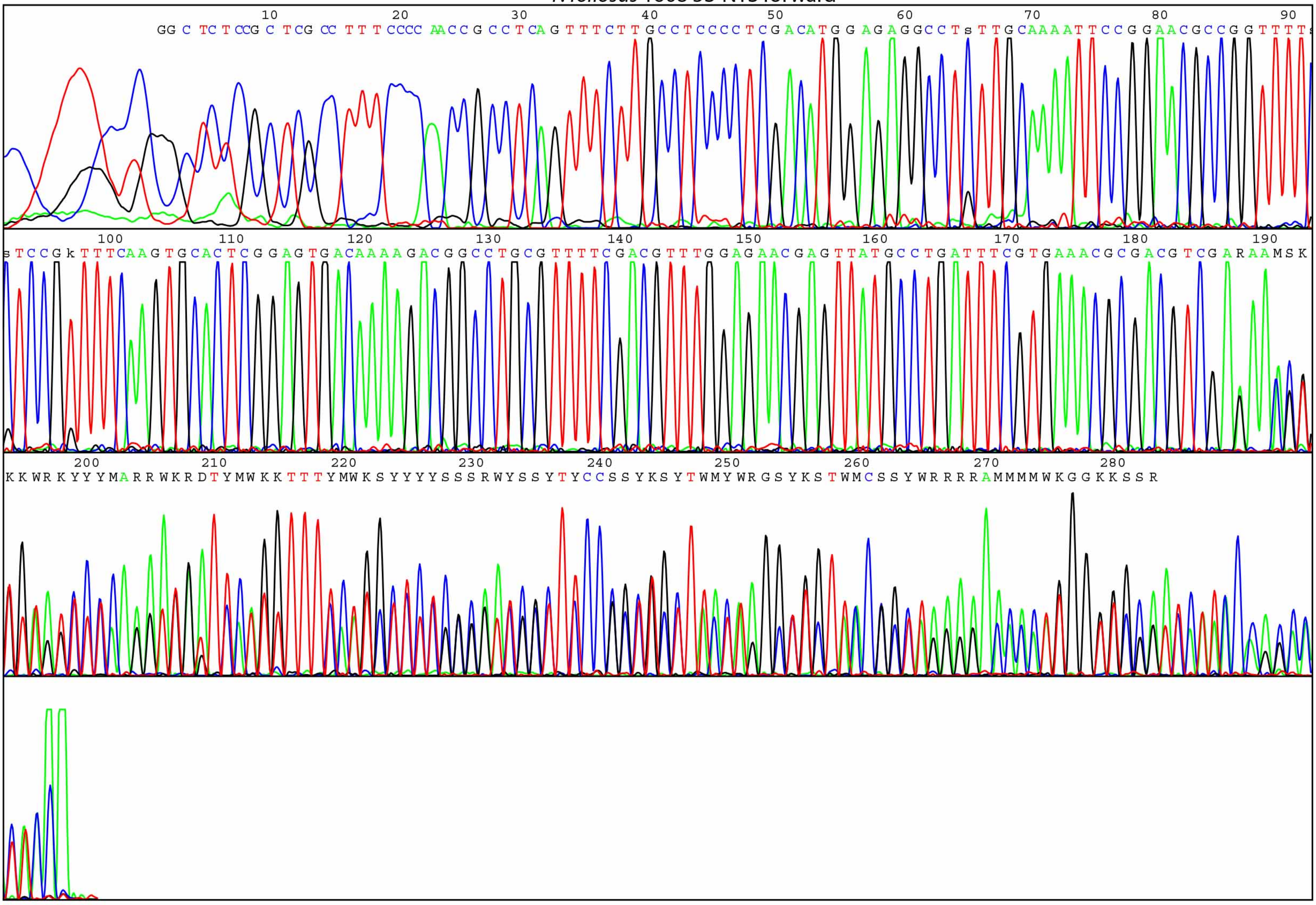

P. foliosus 1608 5S-NTS reverse

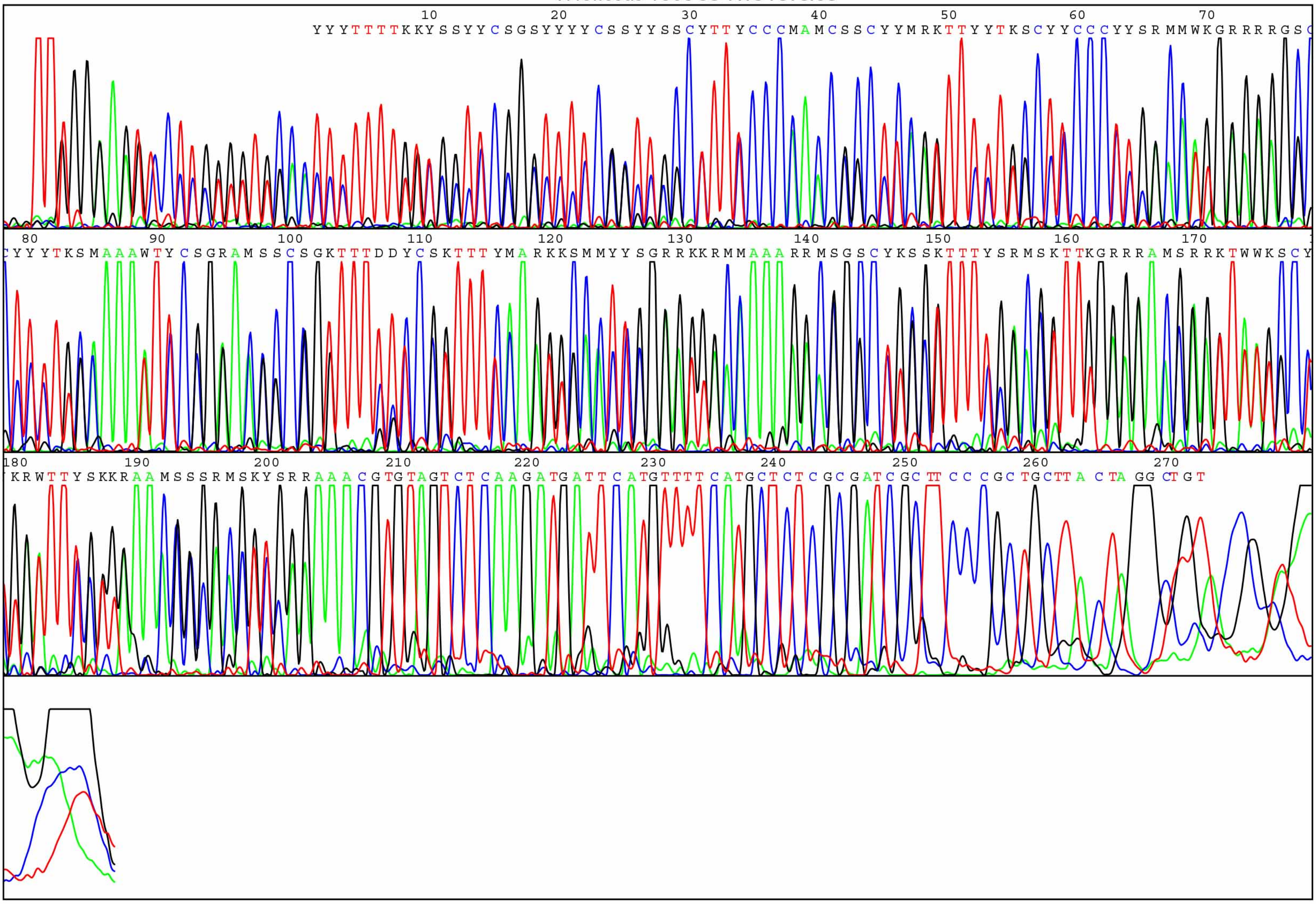

P. pusillus 1712 5S-NTS forward

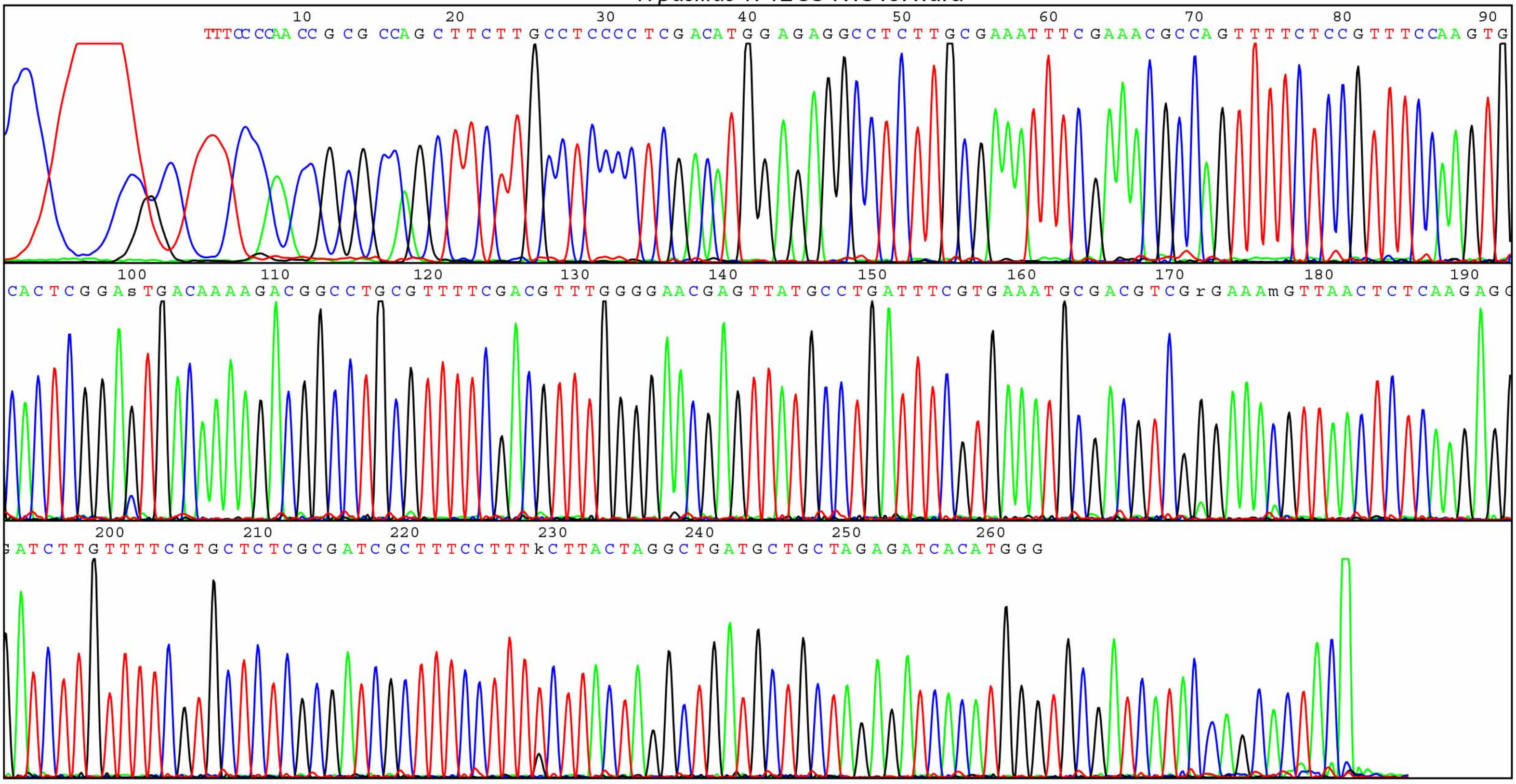

P. pusillus 1712 5S-NTS reverse

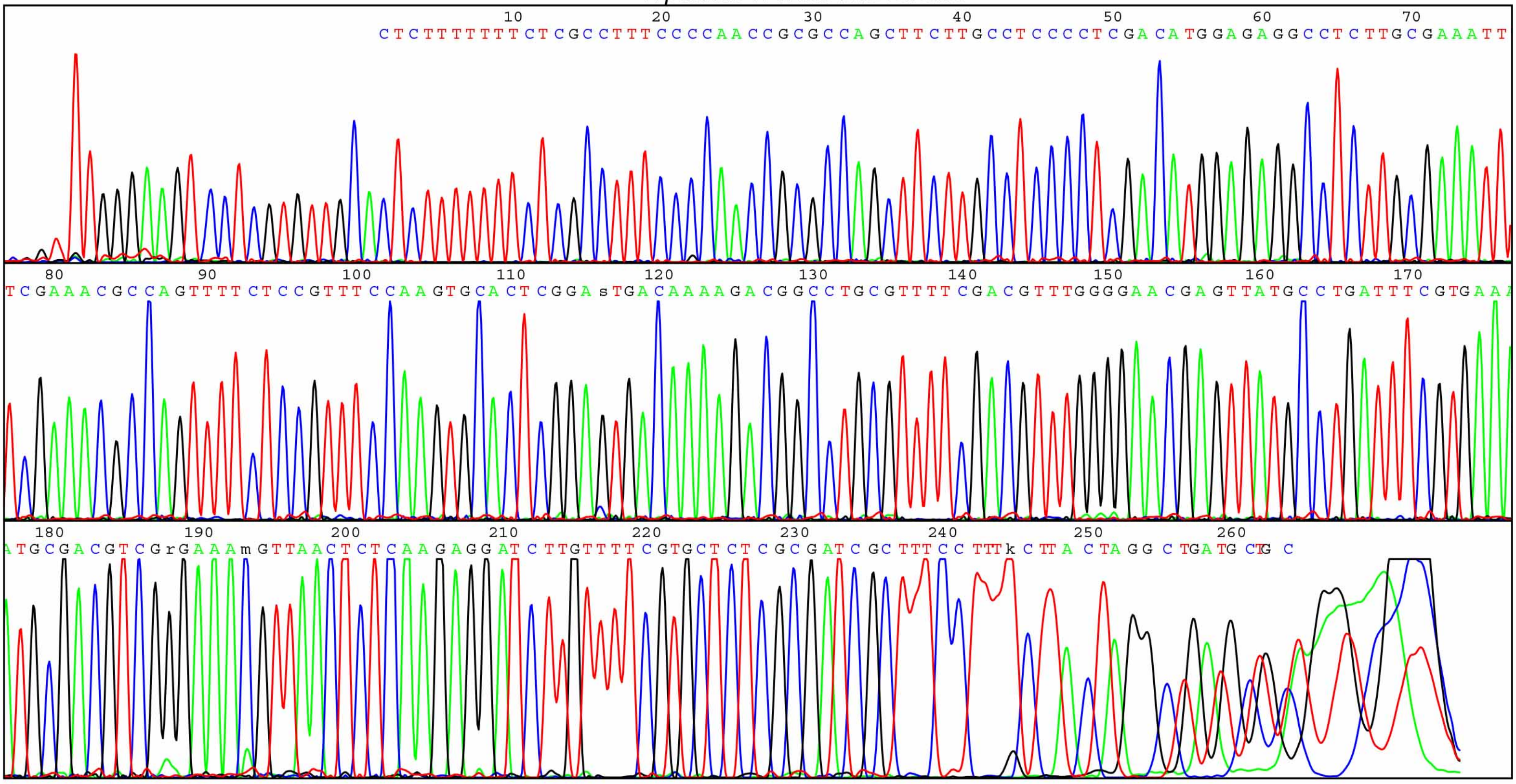

P. berchtoldii 1641 5S-NTS forward

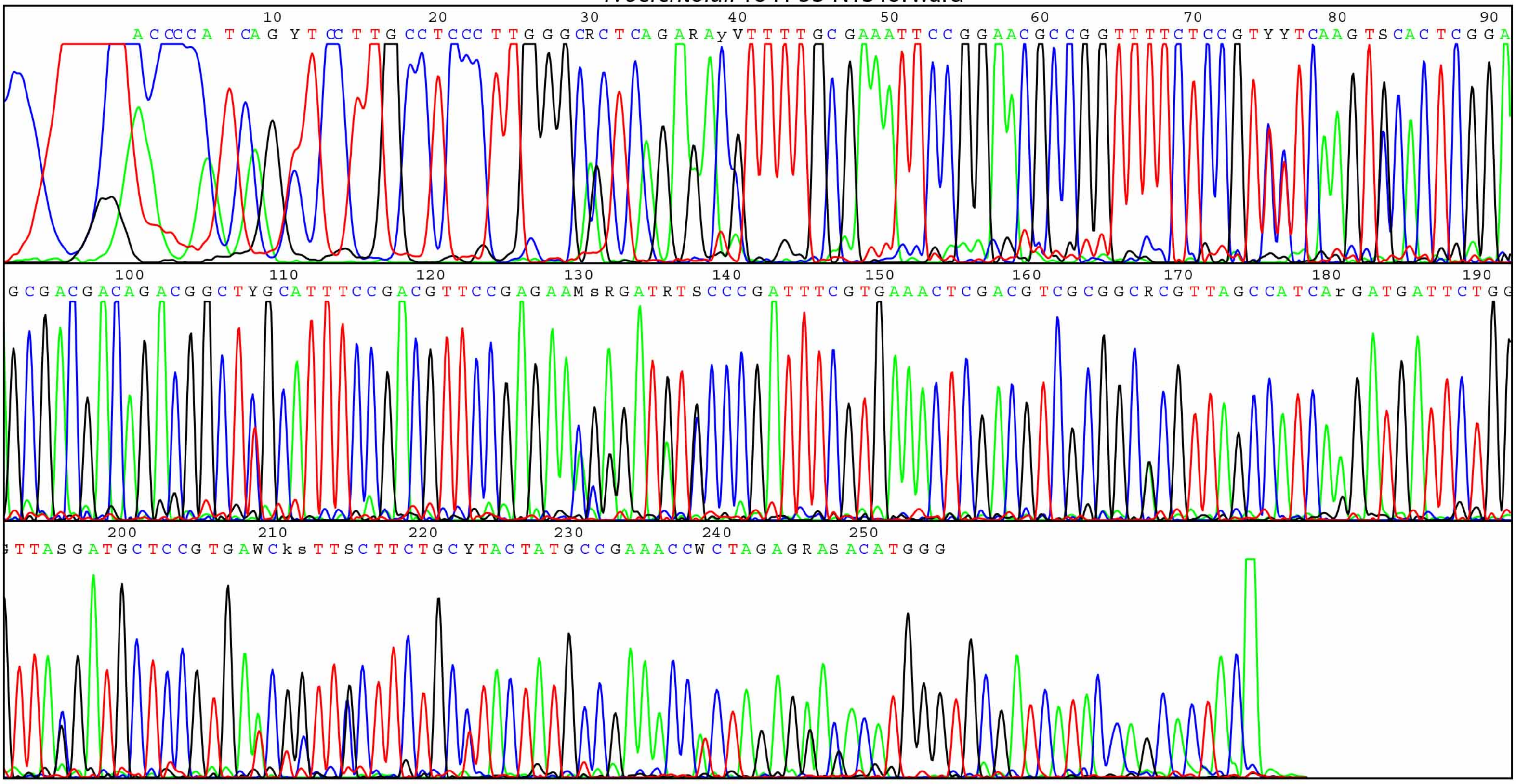

P. berchtoldii 1641 5S-NTS reverse

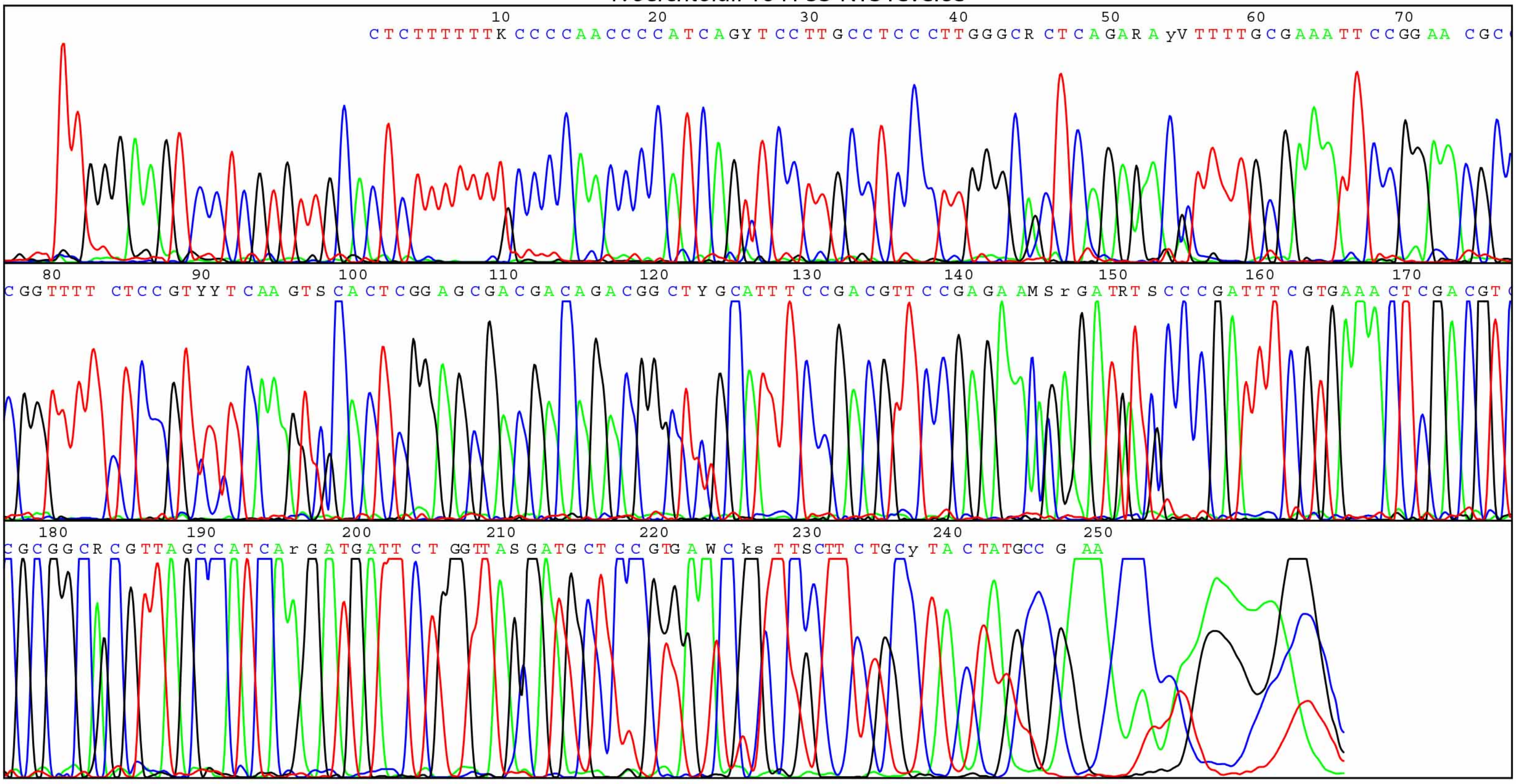

P. illinoensis 1983 5S-NTS forward

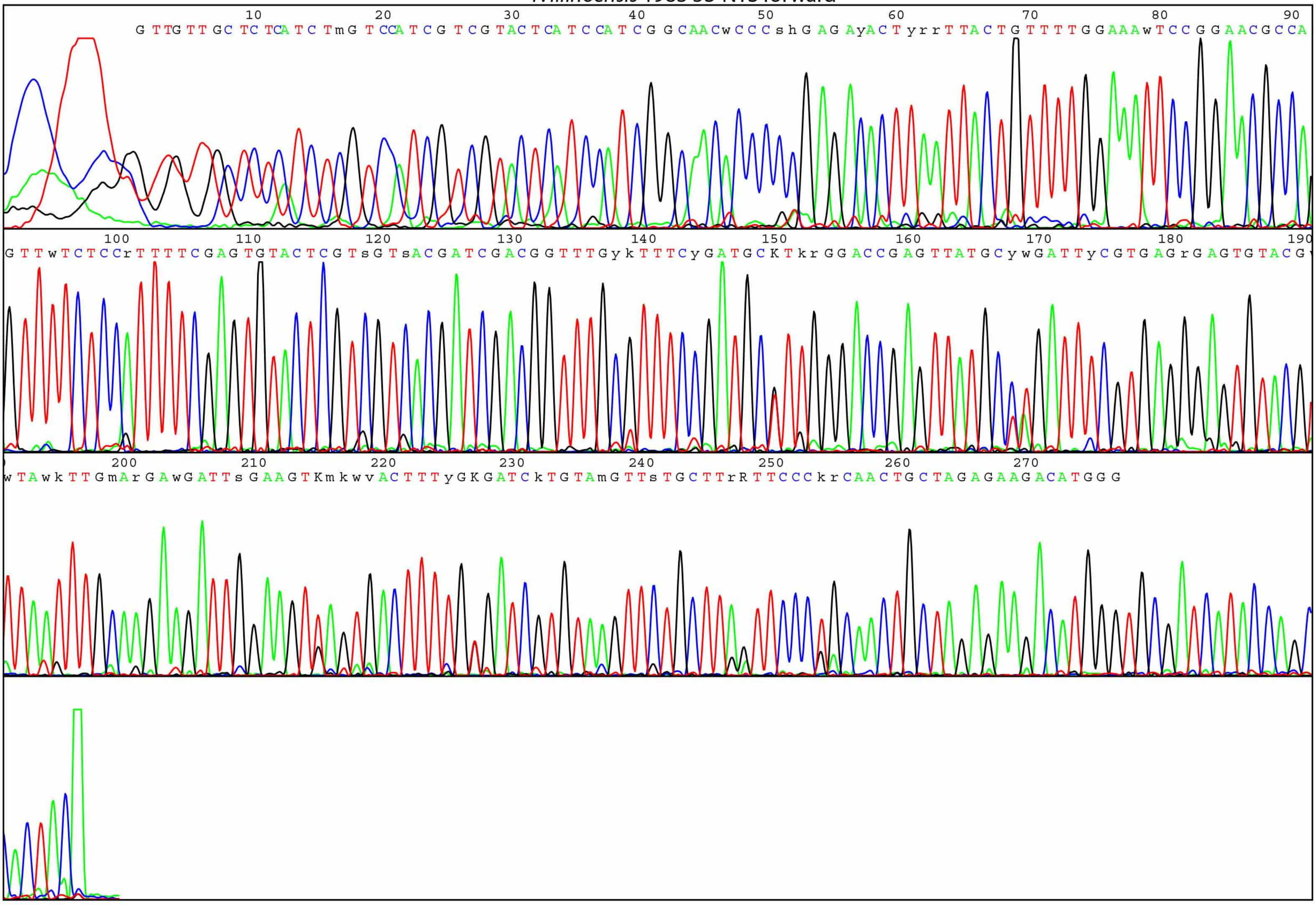

P. illinoensis 1983 5S-NTS reverse

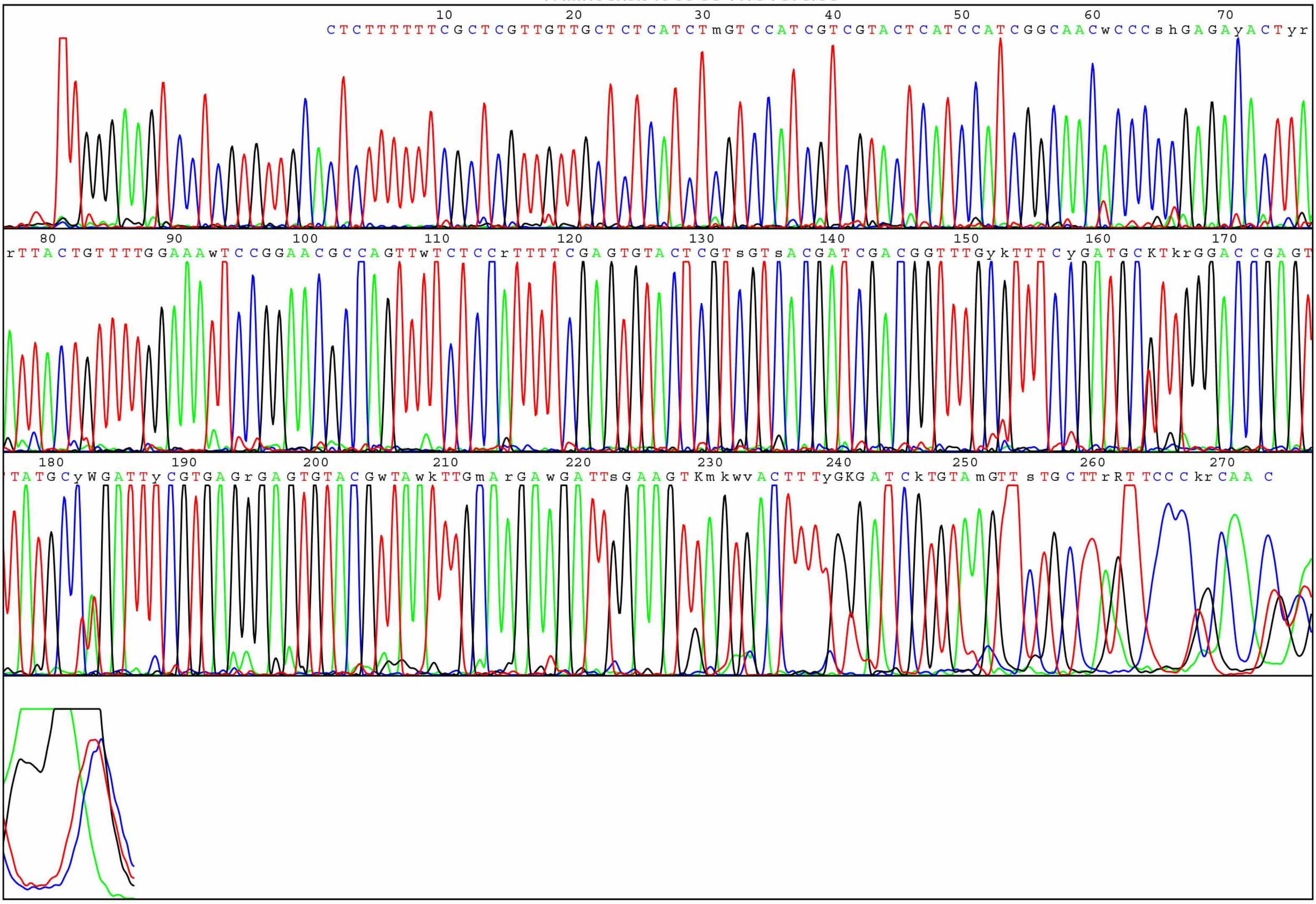

P. nodosus 2284 5S-NTS forward

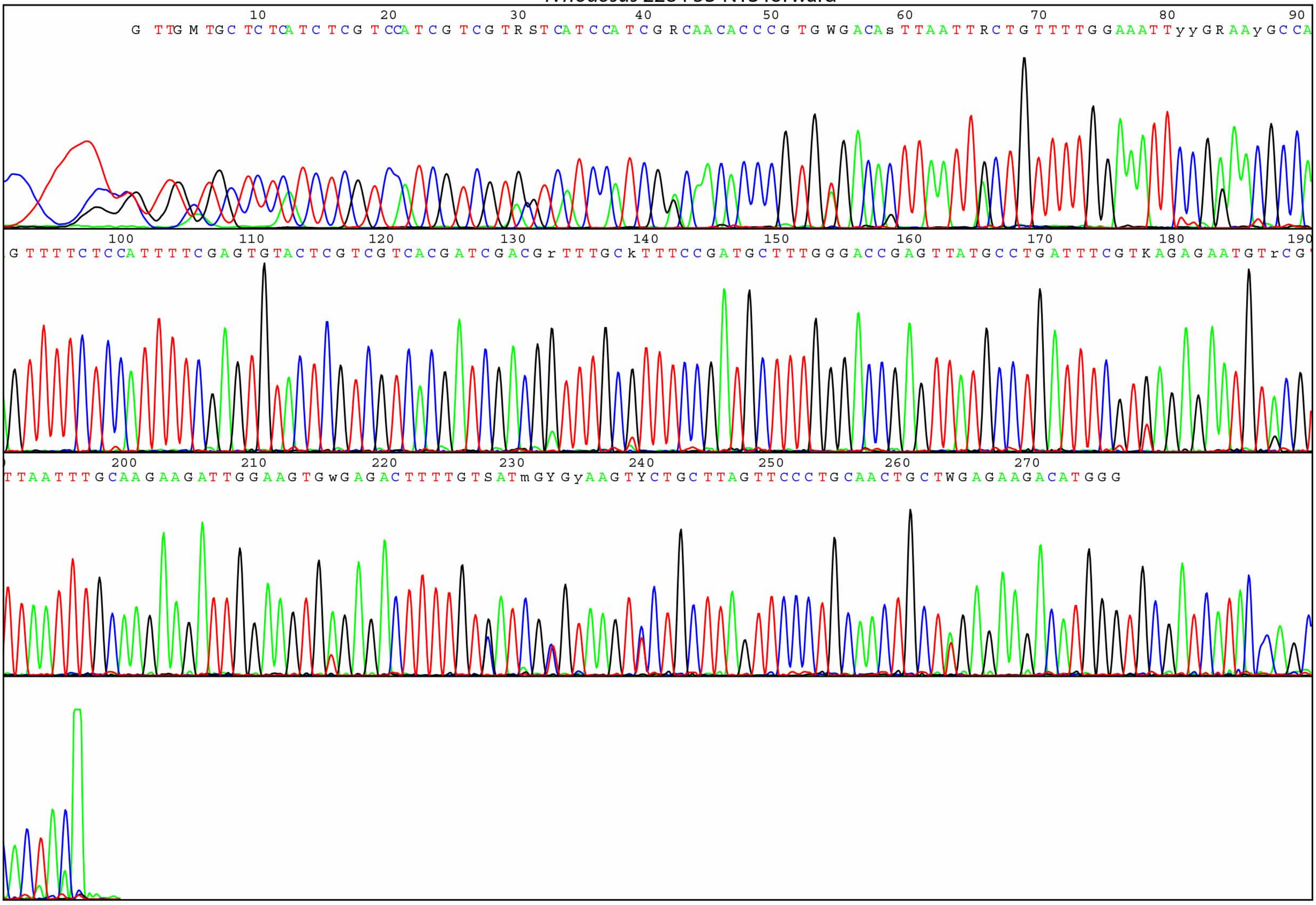

P. nodosus 2284 5S-NTS reverse

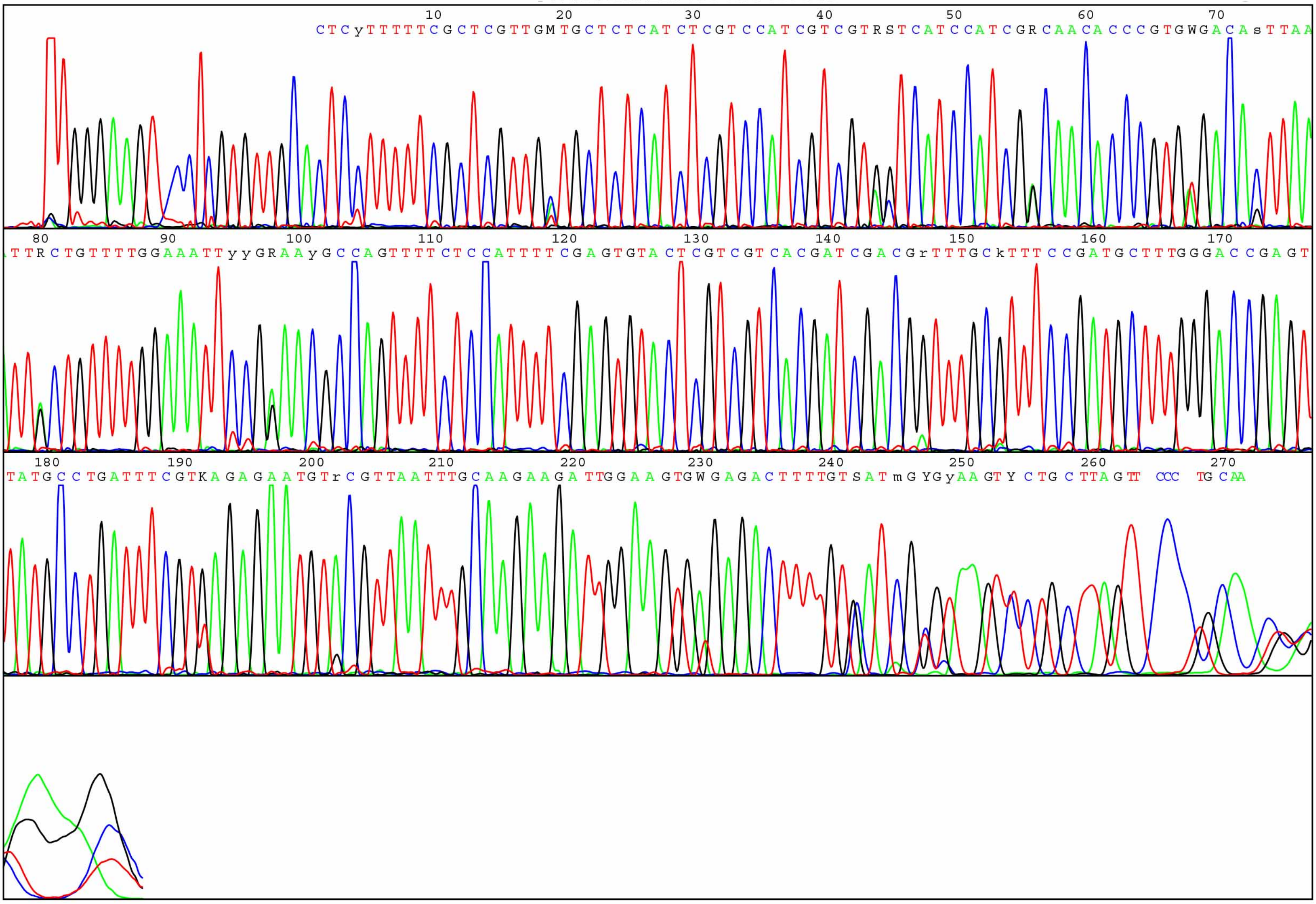

P. 'tepperi' 2364 5S-NTS forward

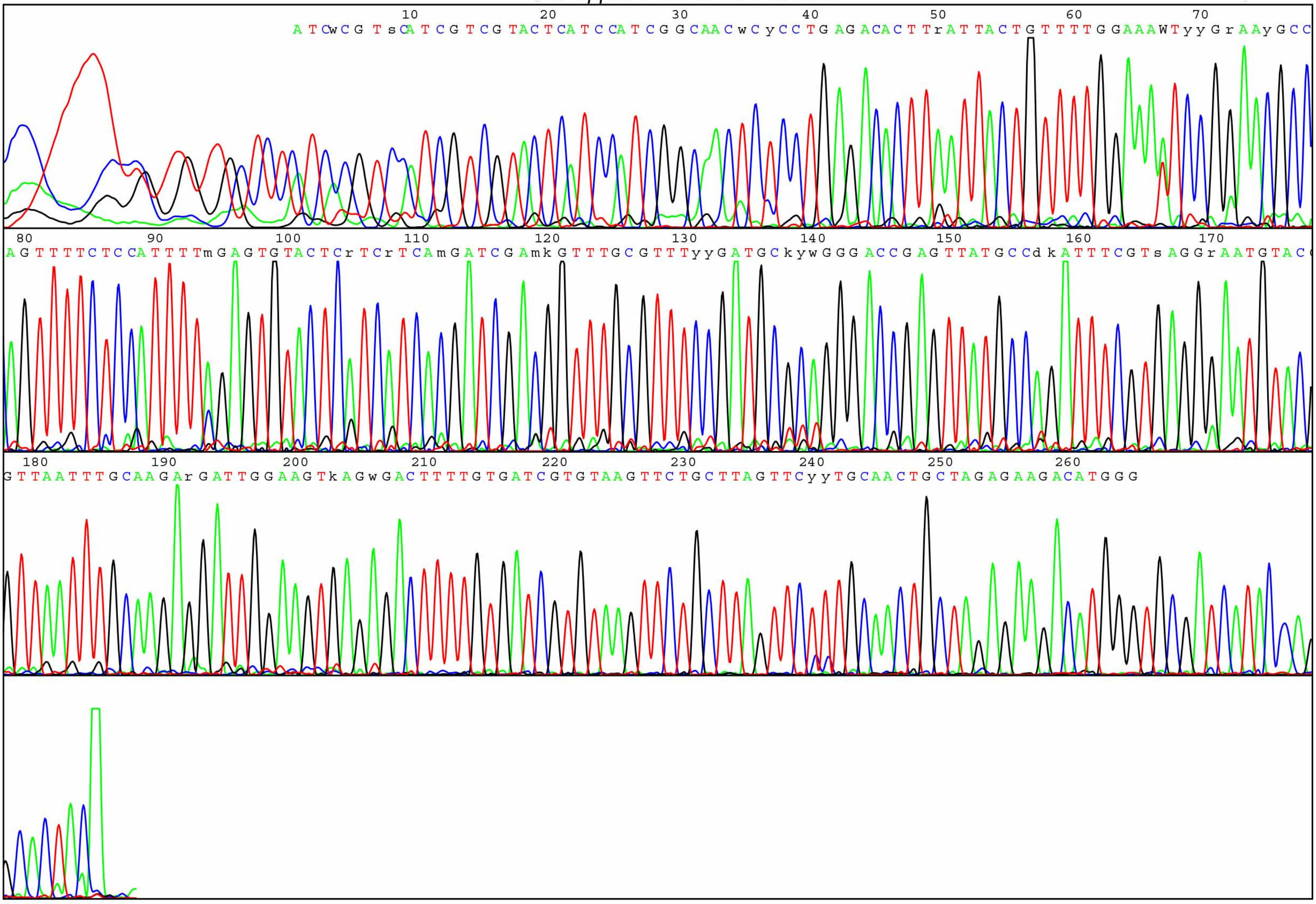

P. 'tepperi' 2364 5S-NTS reverse

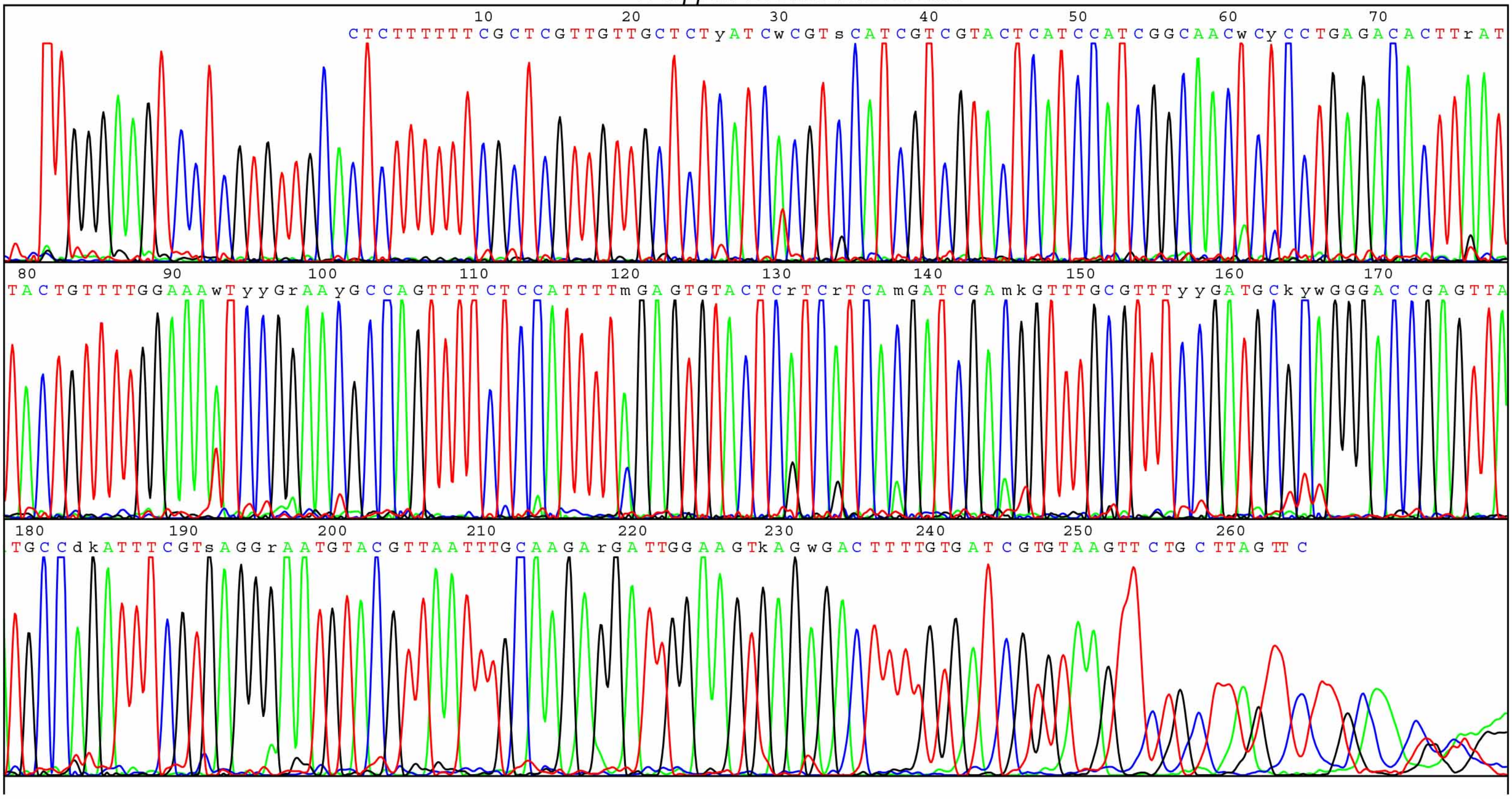

P. distinctus 2675 5S-NTS forward

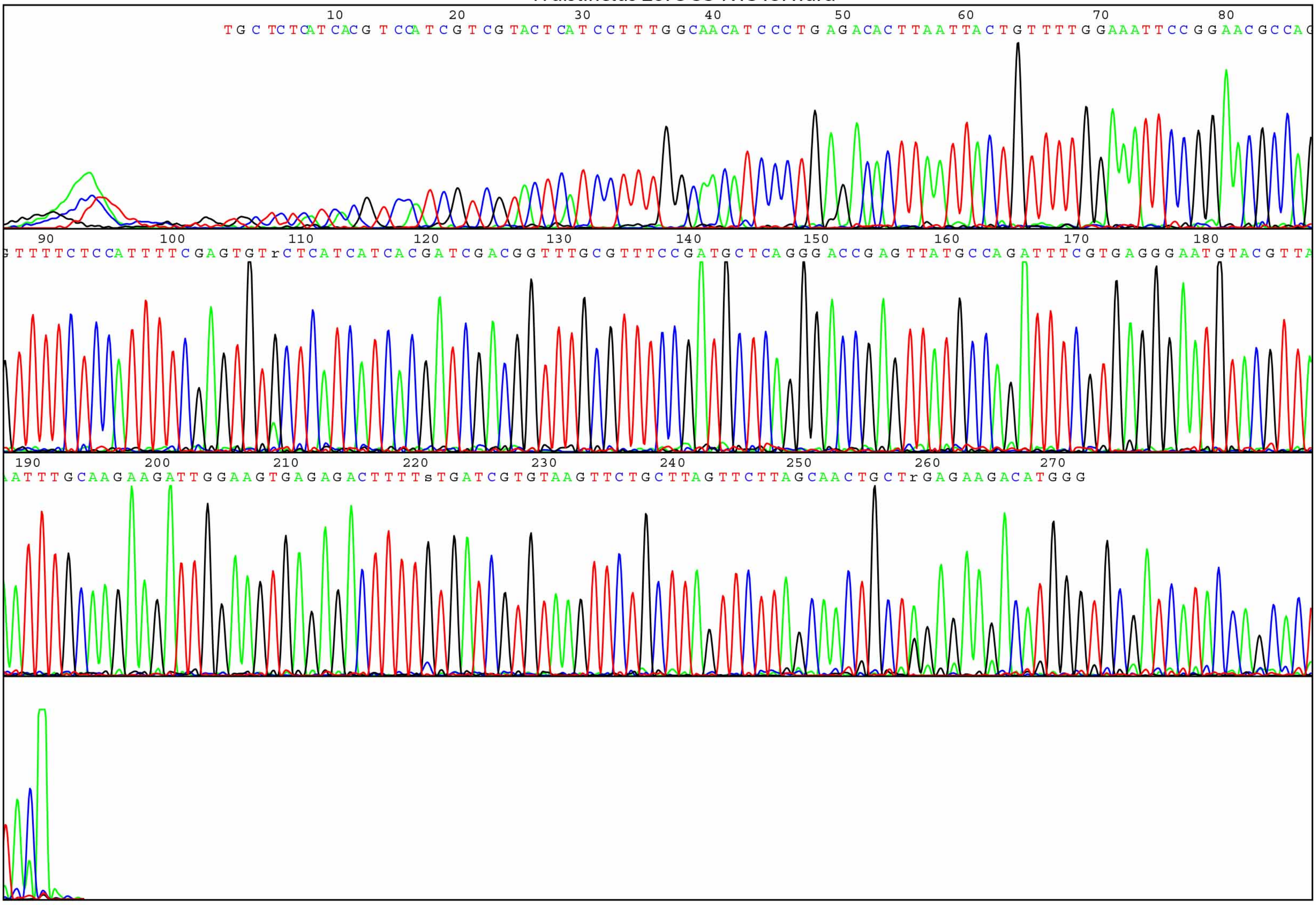

P. distinctus 2675 5S-NTS reverse

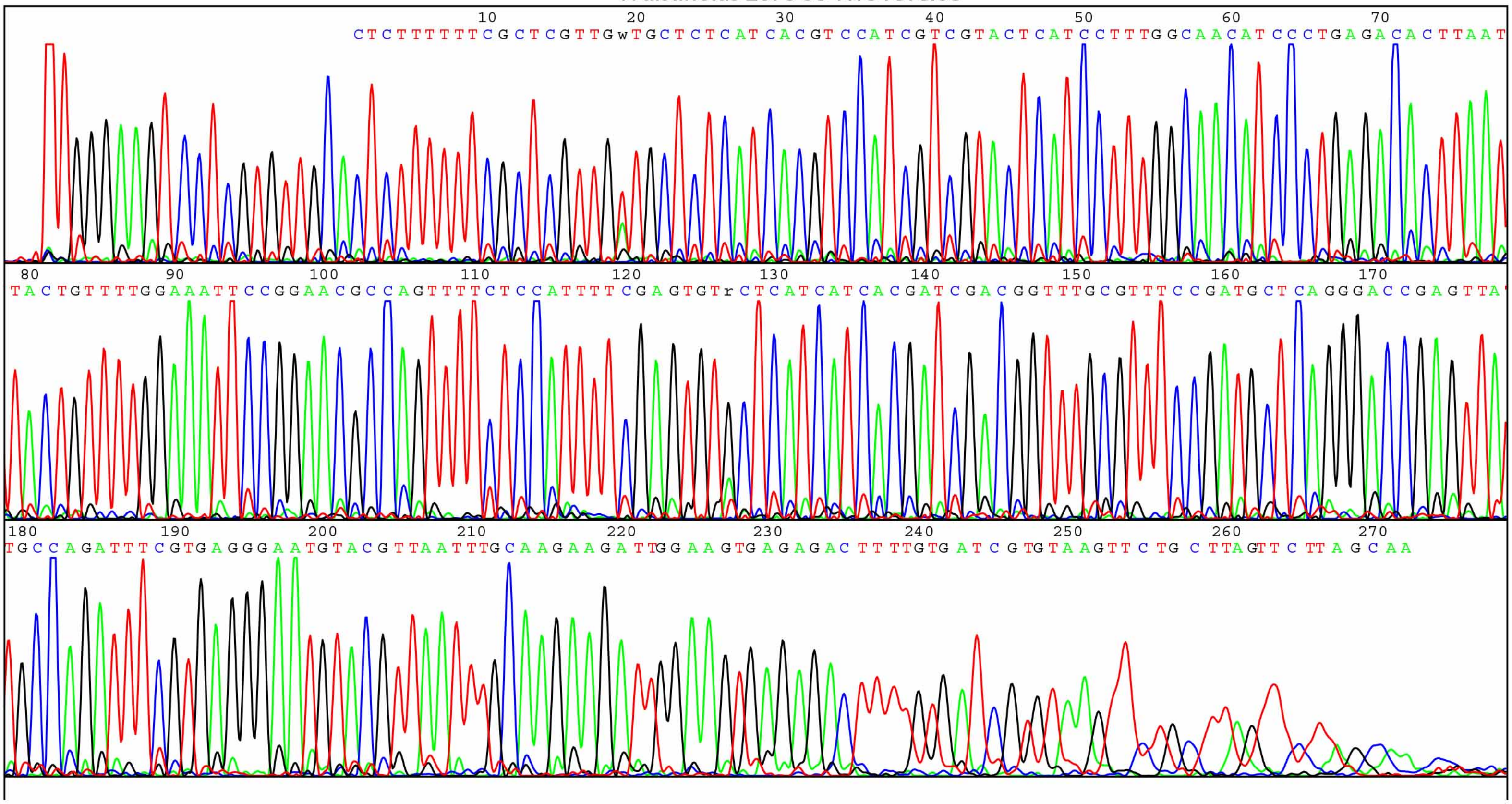

P. natans 1756 5S-NTS forward

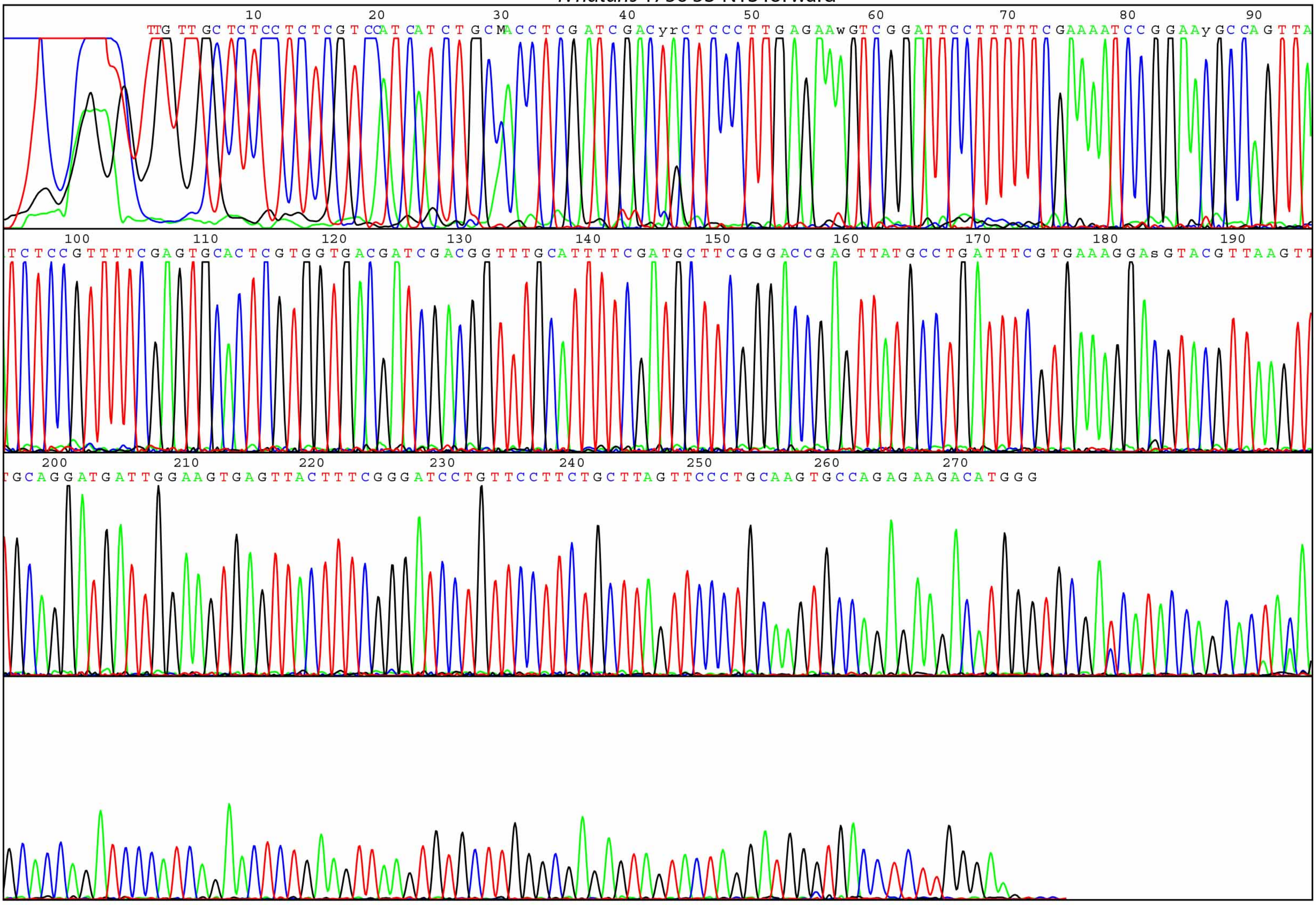

P. natans 1756 5S-NTS reverse

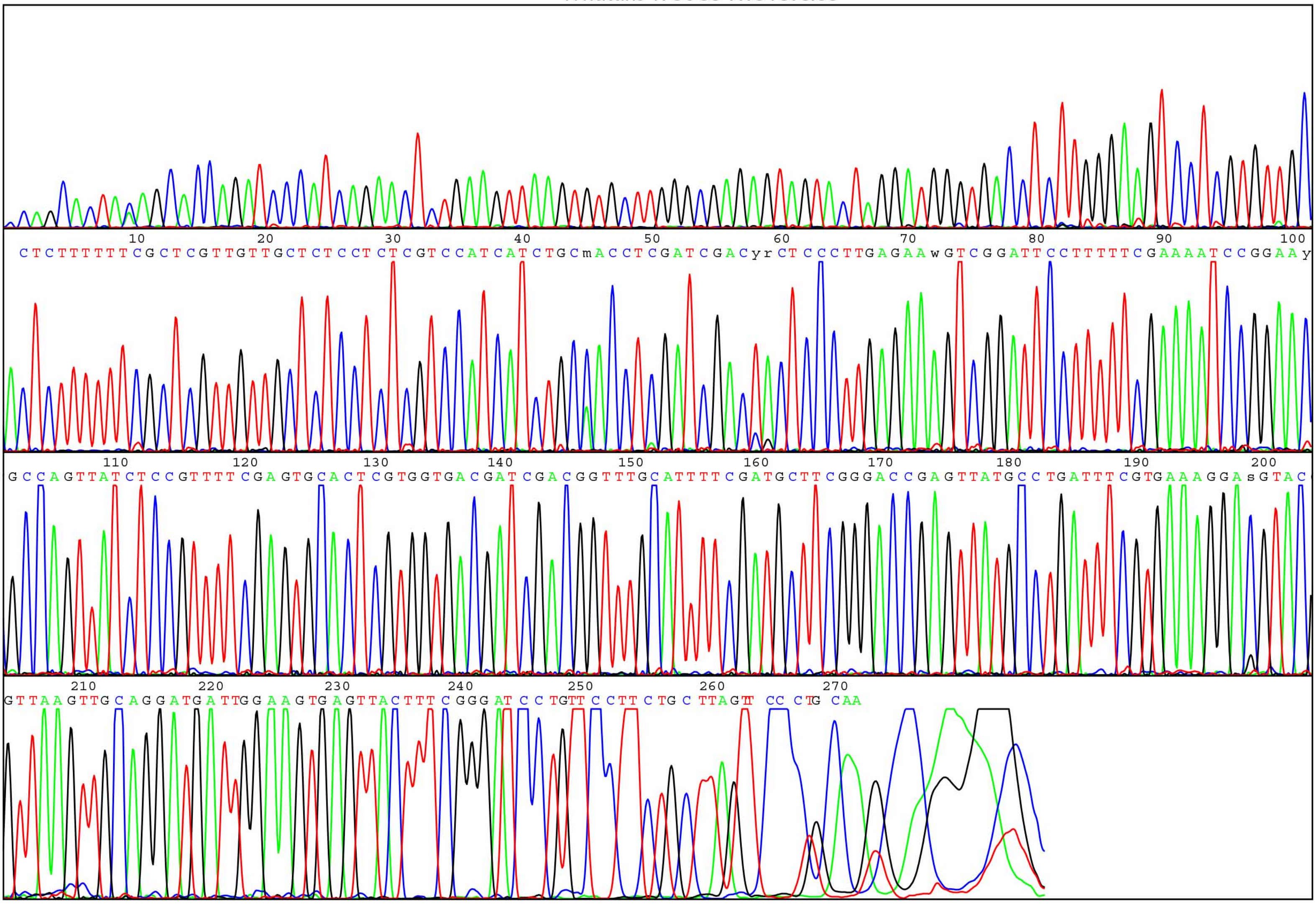

P. oakesianus 1628 5S-NTS forward

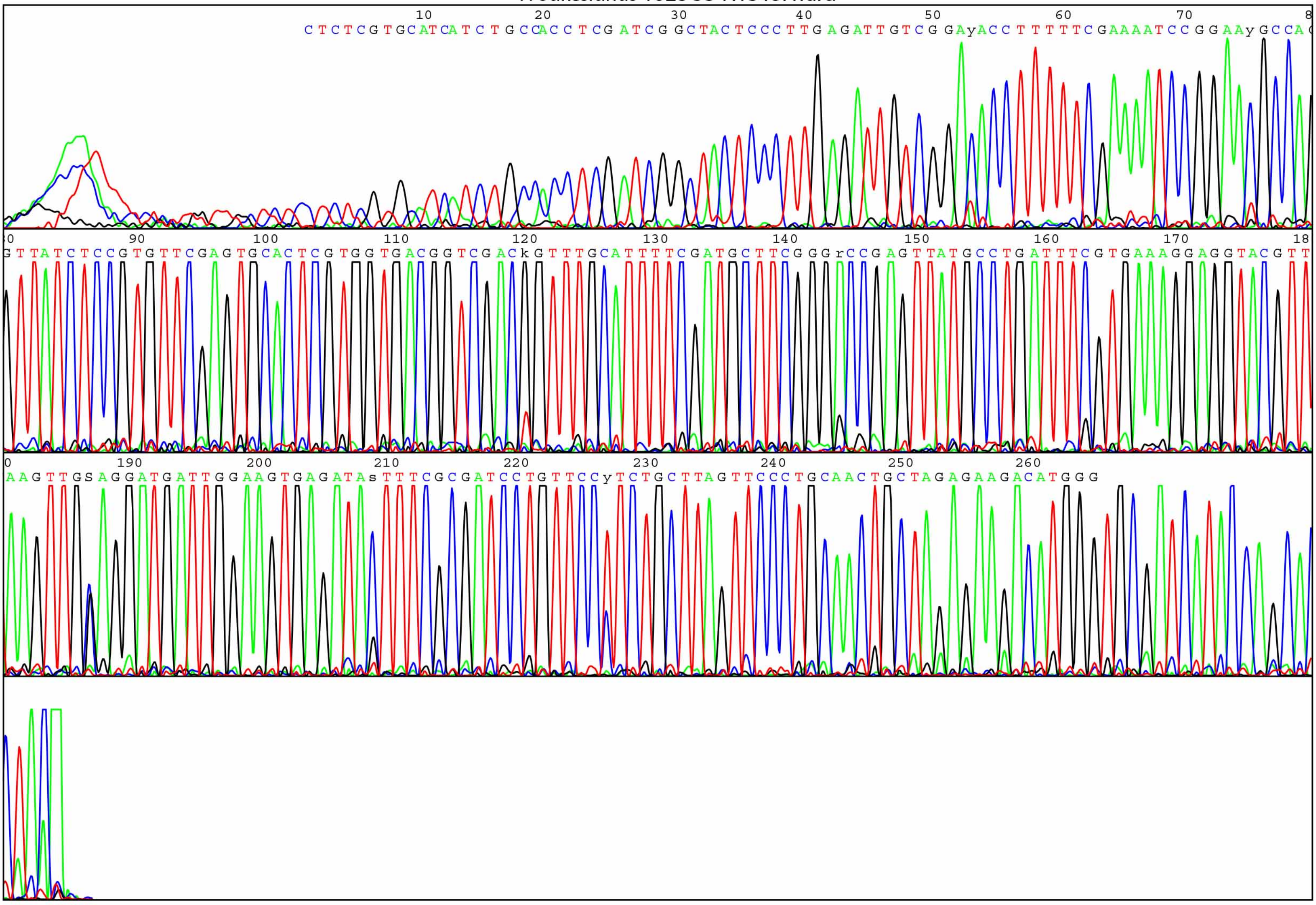

P. oakesianus 1628 5S-NTS reverse

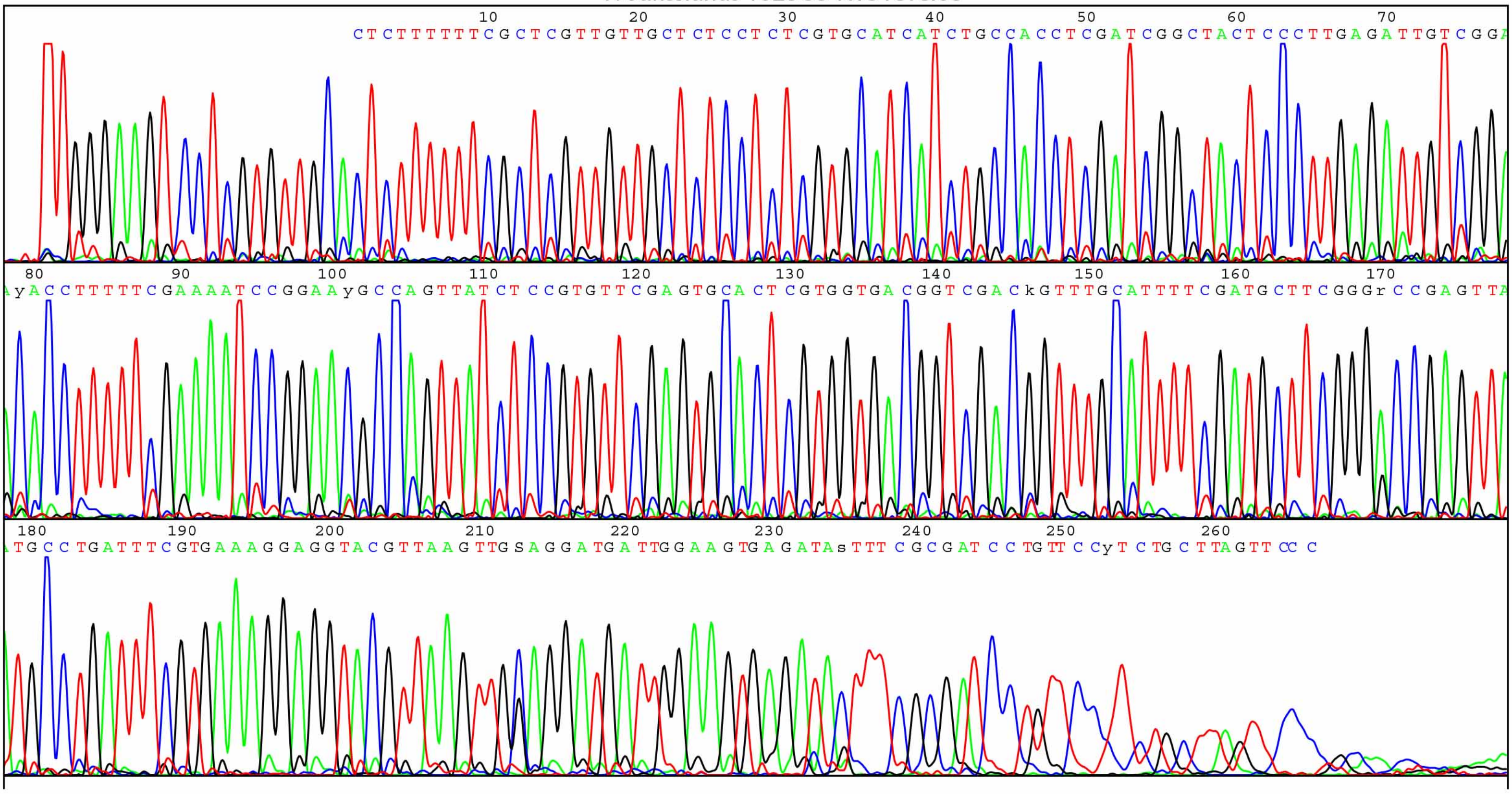

P. x floridanus 2536 5S-NTS forward

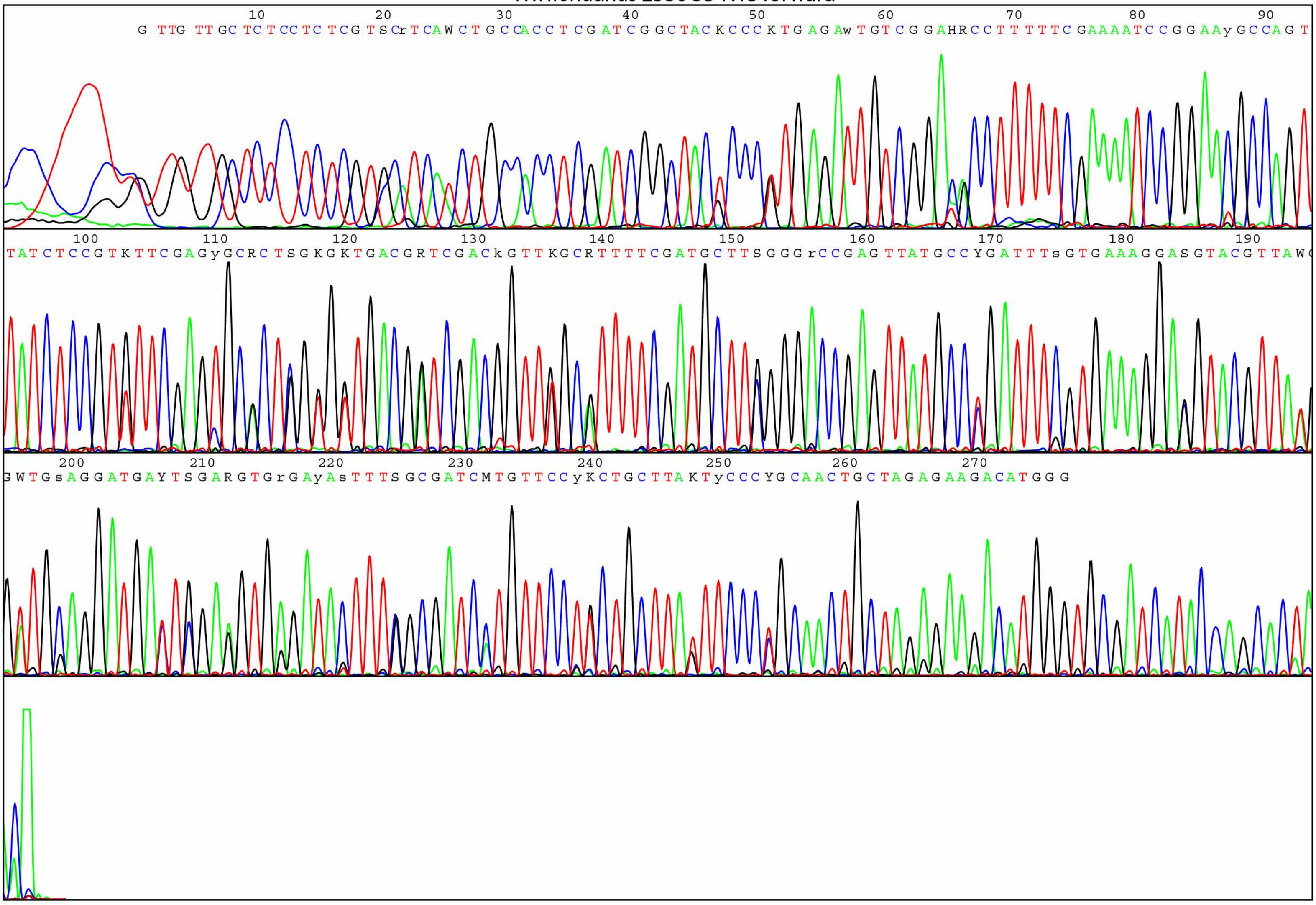

P. x floridanus 2536 5S-NTS reverse

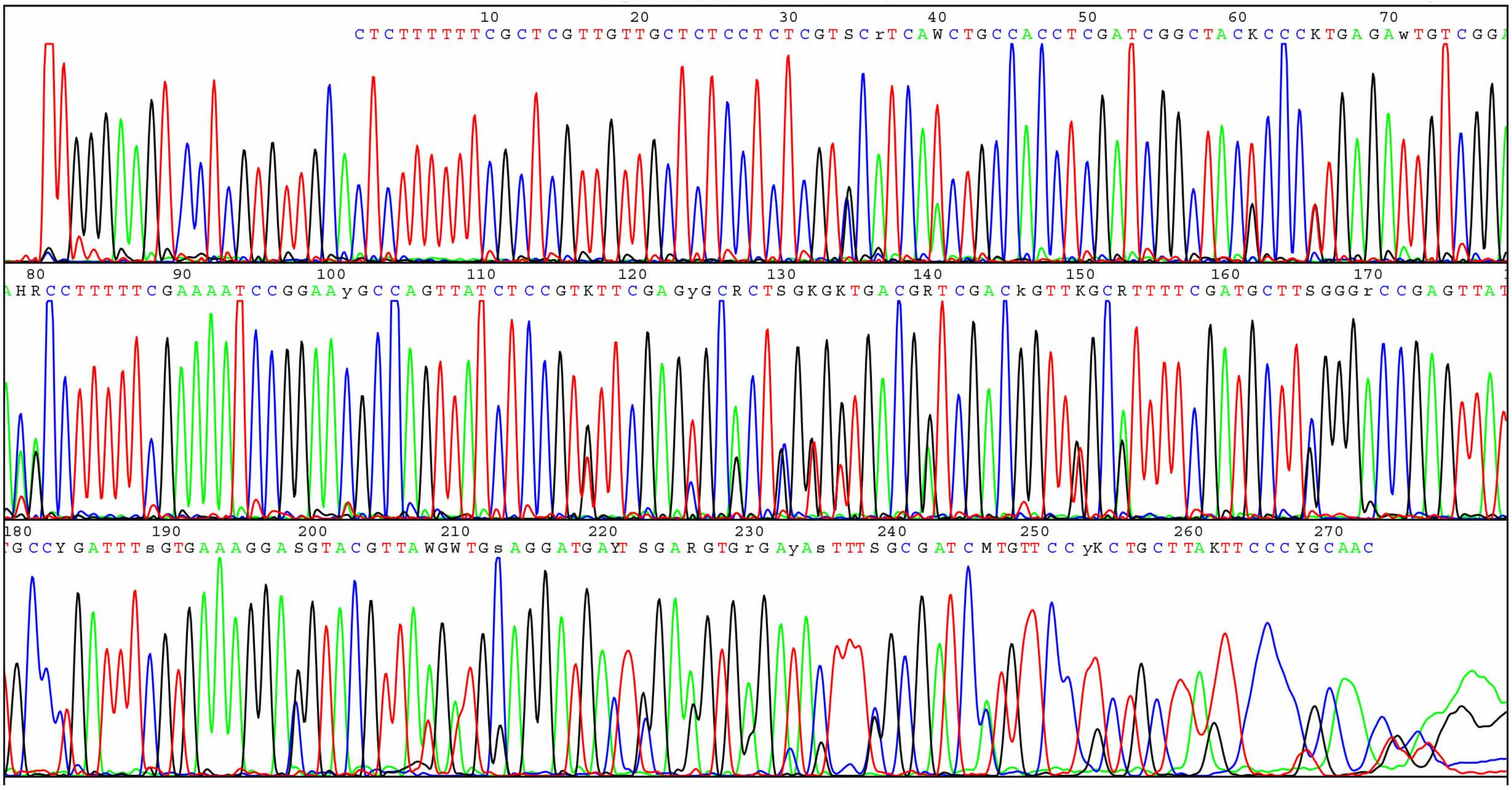

P. pulcher 1681 5S-NTS forward

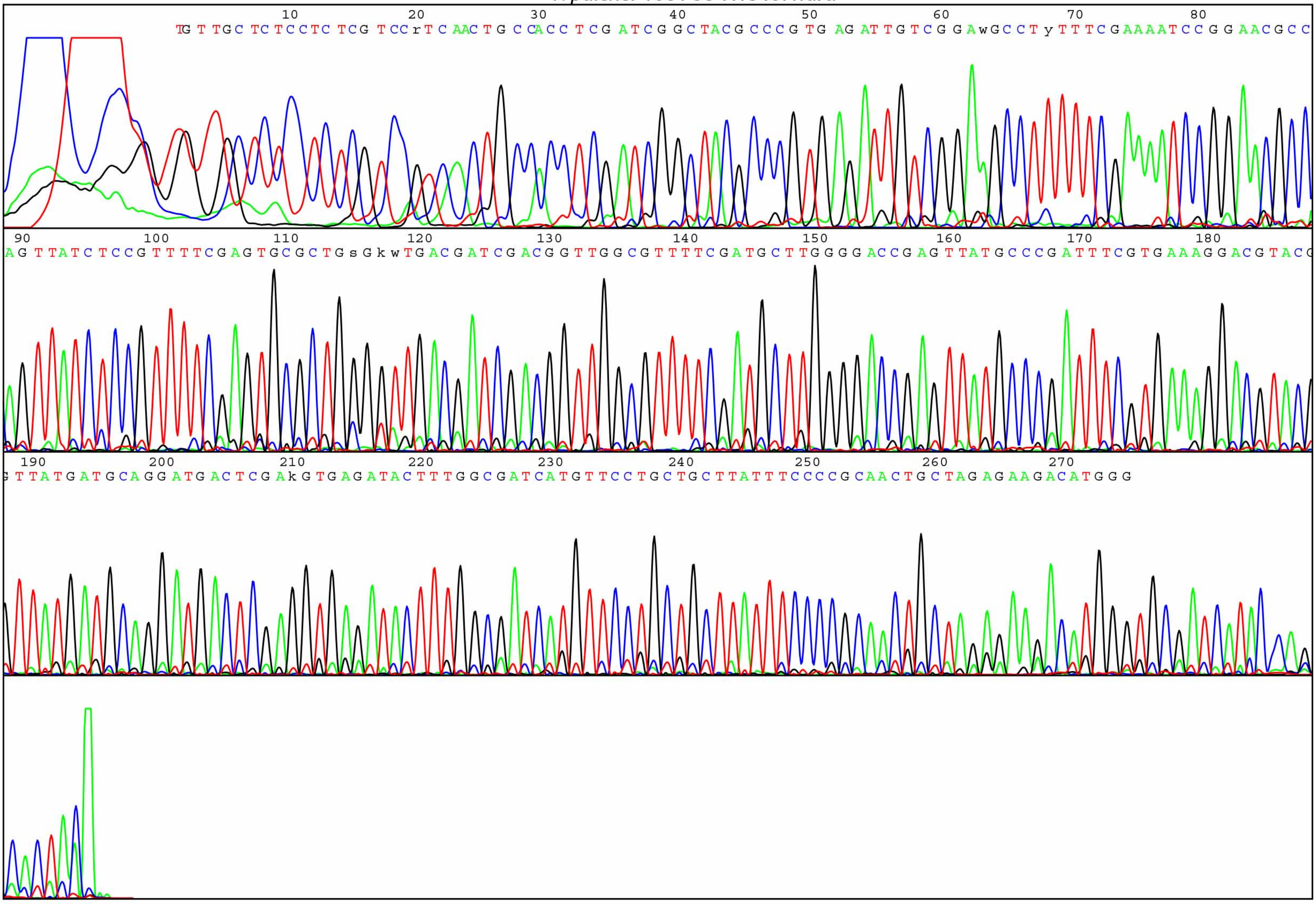

P. pulcher 1681 5S-NTS reverse

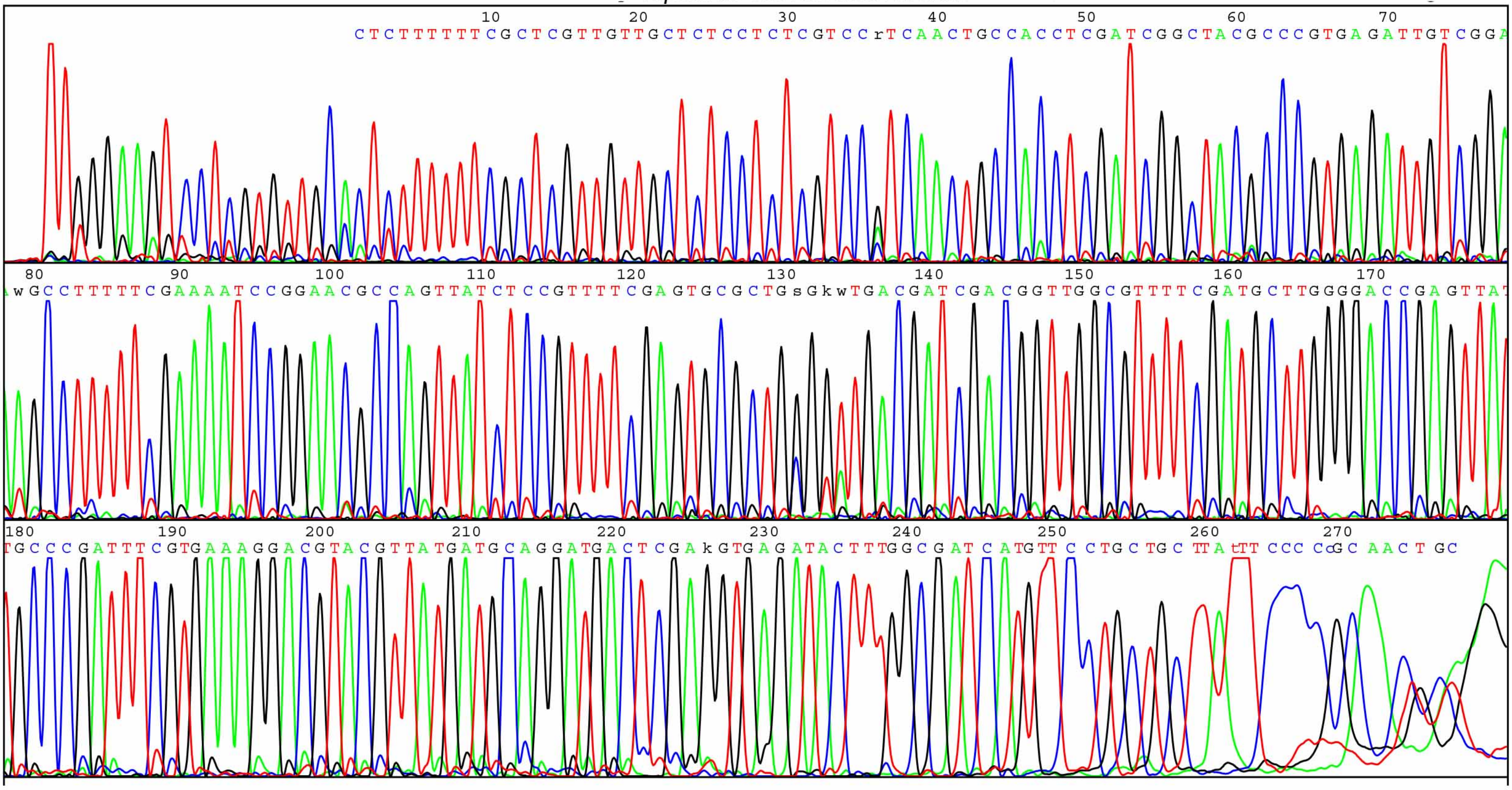

P. amplifolius 2642 5S-NTS forward

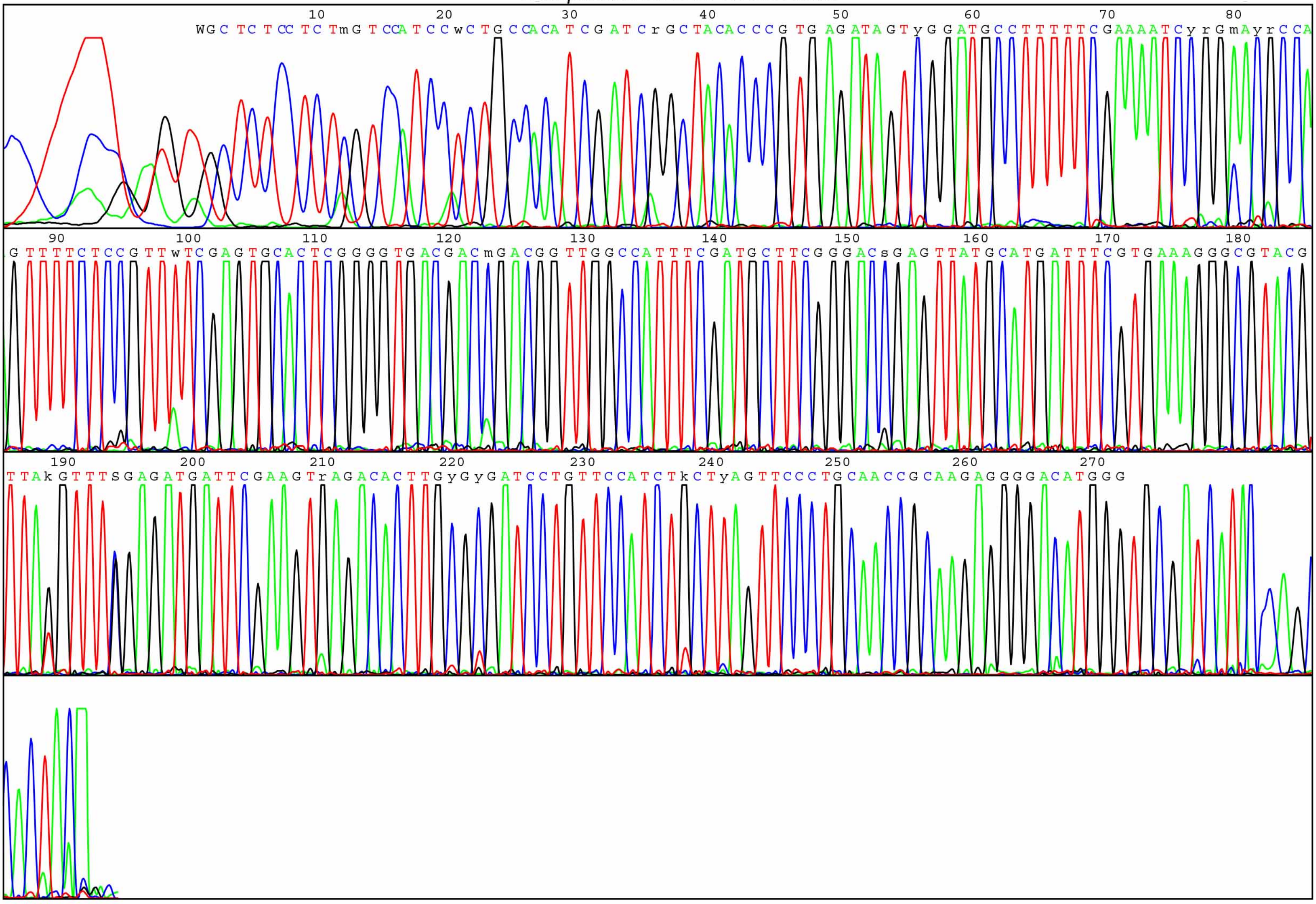

P. amplifolius 2642 5S-NTS reverse

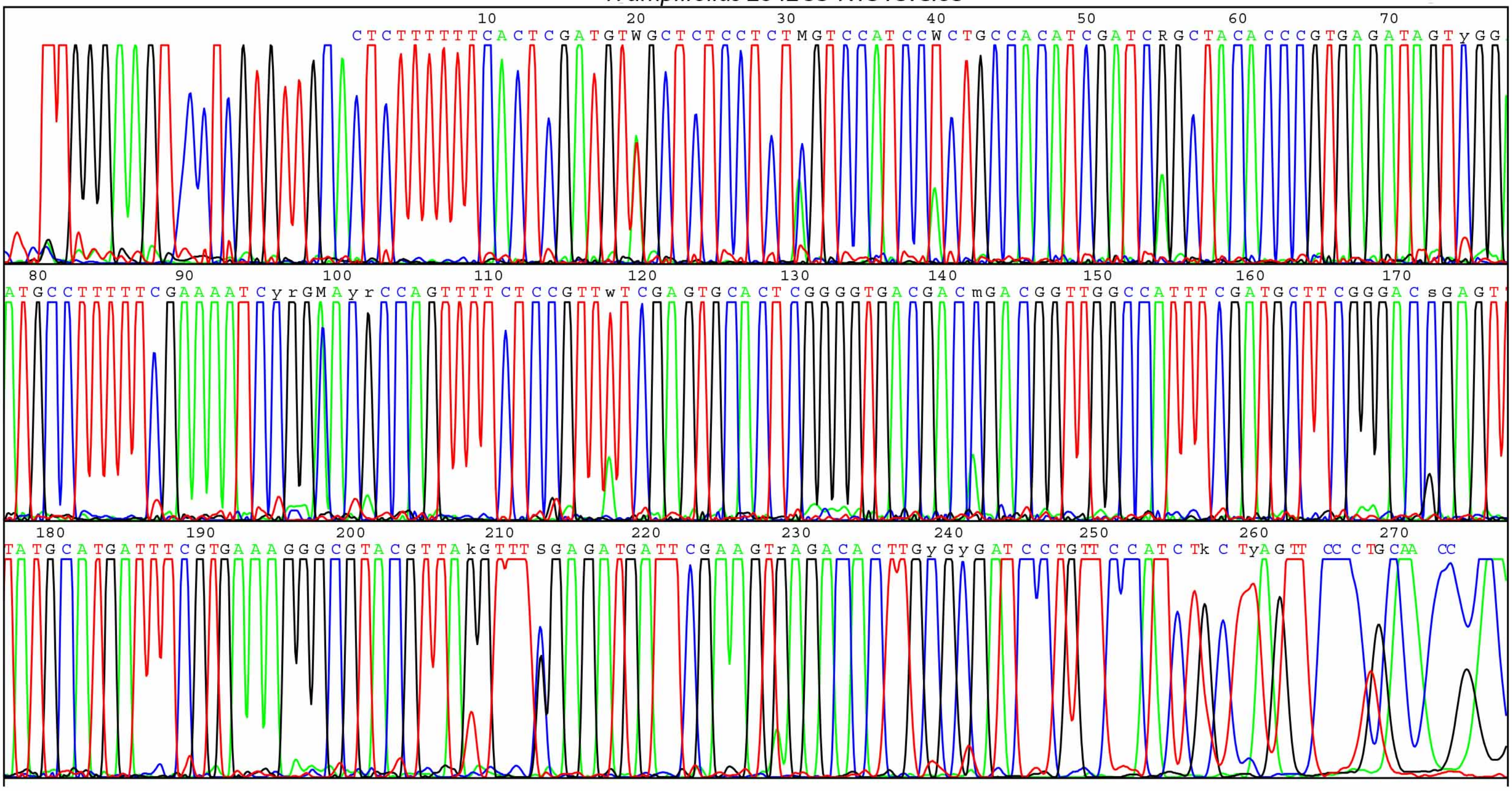

Supplement: S2 Fig — (PDF) [file pone.0195241.s002.pdf]
