# Supplementary material for: The endangered Florida pondweed (Potamogeton floridanus) is a hybrid: Why we need to understand biodiversity thoroughly
Source: PLoS One. 2018 Apr 2;13(4):e0195241. doi: 10.1371/journal.pone.0195241 (PMC5880373; doi:10.1371/journal.pone.0195241)
Supplement: S1 Table — (PDF) [file pone.0195241.s003.pdf]

Supplementary Table S1: Polymorphisms in direct and major 5S-NTS sequences

Species	Identifier or sequence from Lindqvist et al. (2006)	Number of polymorphisms in direct sequence / polymorphisms in major sequence
P. diversifolius	1770 (1)	1 / 0
	1849 (2)	8 / 1
	DQ786488	n.d. / 0
	DQ786489	n.d. / 0
P. foliosus	1608	4 + 1 indel / 1
	DQ786494	n.d. / 0
P. pusillus	1712	4 / 0
	DQ786503[1]	n.d. / 0
	DQ786504[1]	n.d. / 0
	DQ786505[1]	n.d. / 0
P. berchtoldii	1641	26 / 14
P. illinoensis	1983	44 / 3
	DQ786466 (1)	n.d. / 20
	DQ786467 (2) [2]	n.d. / 13
P. nodosus	2284	23 / 9
	DQ786471	n.d. / 1
	DQ786472	n.d. / 2
P. tepperi	2364	31 / 0
	DQ786468	n.d. / 0
	DQ786469	n.d. / 1
P. distinctus	2675	4 / 0
	DQ786470	n.d. / 0
P. natans	1756	6 / 0
	DQ786480	n.d. / 1
P. oakesianus	1628	7 / 1
	DQ786479	n.d. / 0
P. floridanus	2536	32 / 27
	DQ786478	n.d. / 10
P. pulcher	1681	7 / 0
	DQ786475[3]	n.d. / 8
P. amplifolius	2642	20 / 2
	DQ786476	n.d. / 0
	DQ786477	n.d. / 1

[1] actually *P. berchtoldii*
[2] most probably a hybrid with a species not included here
[3] actually a hybrid *P. pulcher* × *P. amplifolius* (see Fig 2)
n.d. – not determined
